# Comparative Genomics of Pathogenic *Clavibacter michiganensis* subsp. *michiganensis* Strains from Chile Reveals Potential Virulence Features for Tomato Plants

**DOI:** 10.3390/microorganisms8111679

**Published:** 2020-10-29

**Authors:** Valentina Méndez, Miryam Valenzuela, Francisco Salvà-Serra, Daniel Jaén-Luchoro, Ximena Besoain, Edward R. B. Moore, Michael Seeger

**Affiliations:** 1 Molecular Microbiology and Environmental Biotechnology Laboratory, Department of Chemistry and Centro de Biotecnología Daniel Alkalay Lowitt, Universidad Técnica Federico Santa María, Valparaíso 2390123, Chile; mvalenzuelao@yahoo.com; 2 Department of Infectious Diseases, Institute for Biomedicine, Sahlgrenska Academy, University of Gothenburg, 413 46 Gothenburg, Sweden; francisco.salva.serra@gu.se (F.S.-S.); daniel.jaen.luchoro@gu.se (D.J.-L.); erbmoore@ccug.se (E.R.B.M.); 3 Culture Collection University of Gothenburg (CCUG), Sahlgrenska Academy, University of Gothenburg, 413 46 Gothenburg, Sweden; 4 Microbiology, Department of Biology, University of the Balearic Islands, 07122 Palma de Mallorca, Spain; 5 Escuela de Agronomía, Pontificia Universidad Católica de Valparaíso, Quillota 2260000, Chile; ximena.besoain@pucv.cl

**Keywords:** *Clavibacter michiganensis*, *Clavibacter*, bacterial canker, tomato, pathogenicity, virulence, comparative genomics, phylogenomics

## Abstract

The genus *Clavibacter* has been associated largely with plant diseases. The aims of this study were to characterize the genomes and the virulence factors of Chilean *C. michiganensis* subsp. *michiganensis* strains VL527, MSF322 and OP3, and to define their phylogenomic positions within the species, *Clavibacter michiganensis*. VL527 and MSF322 genomes possess 3,396,632 and 3,399,199 bp, respectively, with a pCM2-like plasmid in strain VL527, with pCM1- and pCM2-like plasmids in strain MSF322. OP3 genome is composed of a chromosome and three plasmids (including pCM1- and pCM2-like plasmids) of 3,466,104 bp. Genomic analyses confirmed the phylogenetic relationships of the Chilean strains among *C.* *michiganensis* subsp. *michiganensis* and showed their low genomic diversity. Different virulence levels in tomato plants were observable. Phylogenetic analyses of the virulence factors revealed that the *pelA1* gene (*chp/tomA* region)—that grouped Chilean strains in three distinct clusters—and proteases and hydrolases encoding genes, exclusive for each of the Chilean strains, may be involved in these observed virulence levels. Based on genomic similarity (ANIm) analyses, a proposal to combine and reclassify *C. michiganensis* subsp. *phaseoli* and subsp. *chilensis* at the species level, as *C. phaseoli* sp. nov., as well as to reclassify *C. michiganensis* subsp. *californiensis* as the species *C*. *californiensis* sp. nov. may be justified.

## 1. Introduction

The common tomato, *Solanum lycopersicum* L. var. *lycopersicum*, one of the most essential vegetable crops world-wide, was domesticated in Mesoamerica from *S. lycopersicum* L. var. *cerasiforme*, that evolved naturally, initially, in Ecuador and, later, in Peru, from the wild, red-fruited *S. pimpinellifolium* L. [1]. *Clavibacter michiganensis* subsp. *michiganensis* belongs to the bacterial family, *Microbacteriaceae*, within the phylum Actinobacteria and is the causal agent of bacterial canker, which is one of the most important diseases of tomatoes in Chile and world-wide with reports of yield losses up to 80% in commercial fields, being declared by the European Union and other countries as a quarantine organism [2]. Whole-genome-based studies [3,4] have led to a recent reorganization of the taxonomy of the species within the genus *Clavibacter*, wherein five of the nine subspecies of *C. michiganensis* have been reclassified as *Clavibacter* species (Table 1). Well-known pathogens include species (previously classified as *C. michiganensis* subspecies) related to diseases of important agricultural crops, including bacterial ring rot of potatoes (*C. sepedonicus* species), wilting and stunting in alfalfa (*C. insidiosus* species), which are quarantined plant pathogens in several countries in America (including Chile), Europe, Asia and Africa. Other species of the *Clavibacter* genus include *C. nebraskensis* and *C. tessellarius* species, which cause wilt and blight of maize, and leaf freckles and spots in wheat, respectively, *C. capsici* species, which is a pathogen of pepper plants, and *C. michiganensis* subsp. *phaseoli* that is a pathogen of beans [5,6,7]. Tomato seed-associated non-pathogenic strains of *C. michiganensis* subsp. *californiensis* and subsp. *chilensis*, have been described as two subpopulations that are phylogenetically related to *C. michiganensis*-like bacteria [8].

The bacterium, *C. michiganensis* subsp. *michiganensis*, invades plant xylem vessels, causing canker on the stem, wilt and discoloration of the leaves and petioles, and lesions in the fruit. Diverse bacterial genes have been described that are implicated in the virulence of this pathogen, including serine proteases and cell wall-degrading enzymes [9,17,18]. Endophytic strains of *C. michiganensis* have been reported to colonize tomato vascular tissue without inducing disease symptoms, also indicating incidences of non-pathogenic strains of *C. michiganensis* [18,19,20]. The genome sequence of the well-characterized pathogen, *C. michiganensis* subsp. *michiganensis* strain NCPPB 382, has been determined and pathogenicity determinants have been described, providing an important reference [17,20]. Genes associated with virulence are located on the chromosome and on two circular plasmids, pCM1 (27.5 kb) and pCM2 (70 kb).

Even with the prevalence and history of tomato bacterial canker in Chile and its impact on agriculture, information about this disease is scarce. In 2018, Valenzuela et al. [21] reported a comparative study of *C. michiganensis* subsp. *michiganensis* strains isolated from different countries, including 25 Chilean strains. A multi-locus sequence analysis (MLSA) and multi-locus sequence typing (MLST) analysis, based on five house-keeping genes, (*atpD*, *dnaK*, *gyrB*, *ppk*, and *recA* [21,22,23]), and multi-locus variable number tandem repeat (VNTR) analysis (MLVA), based on eight VNTRs from the *C. michiganensis* subsp. *michiganensis* genome, demonstrated low genotypic diversity among Chilean strains [21]. The analyses delineated the 25 Chilean *C. michiganensis* subsp. *michiganensis* strains within three different phylogenetic clonal complexes, or clusters: 21 strains in cluster I, one strain in cluster II and three strains in cluster III. *C. michiganensis* subsp. *michiganensis* strain OP3, isolated from the O’Higgins region in central Chile, groups within cluster I, with twenty of the Chilean strains, and with other strains from unknown origin. Strain MSF322, isolated from the Maule region in central Chile, is the only Chilean strain within cluster II, but exhibits phylogenetic relationships with strains from Brazil and Uruguay. Strain VL527, isolated from the Valparaiso region, clusters with two other Chilean strains, as well as six *C. michiganensis* subsp. *michiganensis* strains from Algeria, Belgium, France, the Netherlands, Uruguay, and the USA, in cluster III. The study of Valenzuela et al. [21] established that Chilean *C. michiganensis* subsp. *michiganensis* strains possess low genotypic diversity, which was not expected, due to pervasive seed exchange between Chile and other countries. Despite the relatively low genotypic diversity observed among Chilean strains of *C. michiganensis* subsp. *michiganensis*, distinctly different levels of plant pathogenicity have been observed.

In this study, whole-genome sequencing and comparative genomic analysis were performed on three Chilean *C. michiganensis* subsp. *michiganensis* strains, VL527, MSF322 and OP3, representatives of three different phylogenetic clades, based on the previous MLSA-MLST and VNTR-MLVA studies, and exhibiting different levels of virulence in the tomato. The aims of this study were to characterize the genomes and the genomic virulence factors of Chilean *C. michiganensis* subsp. *michiganensis* strains, VL527, MSF322 and OP3, isolated from tomato plants with bacterial canker, and to define the phylogenomic positions of these strains within the species, *Clavibacter michiganensis*.

## 2. Materials and Methods

### 2.1. Bacterial Strains and Culture Conditions

*C. michiganensis* subsp. *michiganensis* strains VL527 and MSF322, were originally obtained from the Phytopathology Laboratory Culture Collection of Chile (Escuela de Agronomía, Pontificia Universidad Católica de Valparaiso, Chile). Strain OP3 was obtained from the culture collection of the Molecular Microbiology and Environmental Biotechnology Laboratory (Chemistry Department, Universidad Técnica Federico Santa María, Valparaíso, Chile). The three *C. michiganensis* subsp. *michiganensis* strains were deposited in the Culture Collection University of Gothenburg (CCUG, www.ccug.se) with the accession numbers: CCUG 73202 = VL527; CCUG 72101 = MSF322; and CCUG 72296 = OP3. As reported in a previous study, the three strains were isolated (in the years 2005 (MSF322), 2012 (VL527) and 2015 (OP3)) from symptomatic tomato plants from different agricultural production fields in Chile and are representatives of three distinct phylogenetic clusters based on MLSA-MLST and VNTR-MLVA studies [21]. Strains were routinely cultured on yeast–peptone–glucose agar medium (YPGA; 5 g L^−1^ yeast extract, 5 g L^−1^ bactopeptone,10 g L^−1^ glucose, 15 g L^−1^ agar) and incubated for 3 to 7 days at 28 °C.

### 2.2. Pathogenicity Assays in Tomato Seedlings

Seeds of *Solanum lycopersicum* cv. San Pedro were germinated in pots with peat and maintained in a growth chamber at 20–25 °C with a photoperiod of 16 h for 3–4 weeks. For inoculation of the plants with *C. michiganensis* subsp. *michiganensis* under growth chamber conditions, the strains VL527, MSF322, and OP3 from Chilean culture collections were plated on YPGA medium and incubated for 3 days at 28 °C. Suspensions of bacterial strains were prepared in sterile, distilled water at a concentration of 1 × 10^8^ CFU mL^−1^. The concentration of bacterial cells was confirmed, using the serial-dilution plate method [24]. For each bacterial strain, five tomato seedlings were inoculated by puncturing the stem, 1 cm above the cotyledons, with a sterile needle and pipetting 10 µL of bacterial suspension into the wound [24]. Plants inoculated with sterile distilled water were prepared, as negative controls. Disease symptoms were monitored weekly. The assay was repeated three times. Twenty-one days after inoculation, plants were classified, based on disease symptoms, according to the following scale: 0 = no symptoms; 1 = canker at the site of inoculation; 2 = canker extended on the stem; 3 = yellowing and slight wilting; 4 = wilting of all leaves; 5 = dead plant. The disease index (DI), which has been widely used to evaluate virulence levels of plant pathogens [17,18,24], was calculated, using the following formula that was modified from Mc Kinney [25]:

$$DI=\frac{\sum \left(Severity\;rating\times Plant\;No\;at\;that\;rating\right)}{Total\;plant\;No\times Higher\;rating}\times 100$$

### 2.3. Genome Sequencing, Assembly and Annotation

*C. michiganensis* strains VL527 (=CCUG 73202), MSF322 (=CCUG 72101) and OP3 (=CCUG 72296) were cultivated on a tryptic soy agar medium, at 30 °C, overnight. Cells were harvested from the agar medium, suspended in EDTA-saline buffer (0.15 M NaCl, 0.01 M EDTA, pH 8.0) and incubated with 10 mg mL^−1^ lysozyme during 2 h at 37 °C for the lysis of cells. For Illumina sequencing, genomic DNA of strains VL527, MSF322 and OP3 were isolated, using the Wizard Genomic DNA Purification Kit (Promega, Madison, WI, USA). Additionally, for Oxford Nanopore sequencing, high-molecular weight (HMW) DNA of each strain was obtained, using an optimized protocol of a previously described method [26,27]. Paired-end sequencing was performed, using an Illumina HiSeq 4000 system (strains MSF322 and OP3) and a NovaSeq 6000 (strain VL527) (Eurofins Genomics, Konstanz, Germany). Genomic DNA libraries were prepared, using an optimized protocol; standard Illumina adapter sequences were used. The quality of the Illumina reads was assessed, using the CLC Genomics Grid Worker version 11.0.3 (Qiagen Aarhus A/S, Aarhus, Denmark). The Illumina sequence reads of strains VL527, MSF322 and OP3 were trimmed, using Sickle, version 1.33 [28], and screened for sequence reads with a Phred quality score threshold of 30. Additionally, long-read sequencing of the HMW DNA samples was performed, using a MinION Mk101B Sequencer (Oxford Nanopore Technologies, Oxford, UK). Libraries were prepared, using the Rapid Barcoding Kit (SQK-RBK004), and then sequenced, using a FLO-MIN106 vR9.4 flow-cell, with a sequence run of 48 h on MinKNOWN software, version 1.11.5 (Oxford Nanopore Technologies, Oxford, UK). Albacore, versions 2.1.10 (strain VL527) and 2.3.1 (strains MSF322 and OP3), were used for base-calling. The quality of the Oxford Nanopore reads was assessed, using NanoPlot version X (Oxford Nanopore, Oxford, UK). The trimmed subset of Illumina paired-end reads, together with the base-called Nanopore reads, were used to generate hybrid assemblies for the genome sequences of strains VL527, MSF322 and OP3, using Unicycler assembly pipeline, version 0.4.7, with default parameters [29]. For the strain OP3, two additional *de novo* assemblies were performed, using Canu version 1.5 [30] and Flye version 2.4.2 [31]. Additionally, a reference-based assembly was performed, using the tool “Map Reads to Reference” of the CLC Genomics Workbench version 12.0.3 (Qiagen, Denmark), with default parameters and with the options, “Create stand-alone read mappings” and “Create report”, selected, applying the Illumina reads of OP3 strain for mapping and the genome sequence of *C. michiganensis* strain NCPPB 382 as a reference. Subsequently, the consensus sequence was extracted, using the tool, ”Extract Consensus Sequence”, with default parameters. Assembly assessment and genome sequence statistics were calculated, using QUAST, version 5.0.2 [32]. For taxonomy analysis, the 16S rRNA gene sequences were extracted from the genome assemblies and analyzed, using the “16S-based ID” tool of the EzBiocloud database [33].

The VL527, MSF322 and OP3 genome sequences were annotated, using the Prokaryotic Genome Annotation Pipeline (PGAP) version 4.10 [34]. Alternative annotations were done, using Prokka version 1.14.0 [35]; the proteome of *C. michiganensis* subsp. *michiganensis* strain NCPPB 382 was used as reference [36].

Circular maps of the assembled chromosomes were generated, using the software CGview version 2 [37]. Genes were classified, according to the clusters of orthologous groups (COG) nomenclature [38], and computed, using the HMMER mapping mode of eggNOG-mapper version 1 [39], based on eggNOG 4.5 orthology data [40], prioritizing coverage. Multiple genome alignments were performed, using progressiveMauve with iterative refinement and default seed weight [41]. Genetic maps were generated, using SnapGene software version 5.1.3 (GSL Biotech, San Diego, CA, USA).

### 2.4. Pangenome Analysis of Chilean Strains

Protein sequences of genomes retrieved from GenBank were compared, using BLASTP [42]. Based on the results, homologue searches were performed, with the software, Get_Homologues [43], using two different algorithms: Cluster of orthologous genes triangle (COGT) [44]; and orthologous Markov cluster (OMCL) [45]. Proteins showing, at least, 70% similarity for, at least, 70% of the respective sequence, were considered to be homologues [46]. The total number of clusters comprising the pangenome was determined, using the consensus between the two algorithms. From the total number of clusters, the proteins present in each strain including the reference strain, but absent in the Chilean strains, were removed. The eggNOG and Pfam servers were used for prediction of function for hypothetic proteins.

### 2.5. Phylogenomic Analysis of Strains of C. michiganensis

Genome-to-genome relationships between Chilean *C. michiganensis* subsp. *michiganensis* strains VL527, MSF322 and OP3 and other strains of *C. michiganensis* subsp. *michiganensis* and *Clavibacter* spp. were analyzed through average nucleotide identity based on MUMmer (ANIm), using whole-genome sequence datasets (Table S4) [47]. Pairwise genome comparisons were performed, as implemented in the pyani Python3 module [48]. Heatmaps for the genome comparisons were generated, using the pheatmap version 1.0.8 R package [49]. A total of 46 publicly available genome sequences, from strains identified as *C. michiganensis* in the NCBI (RefSeq) database (February 2019), including the type strains of all nine validly published *C. michiganensis* subspecies, and the genome sequences of 3 Chilean strains of this study, were included for ANIm analysis. A total of 49 strains, listed as *C. michiganensis* subsp. *michiganensis* (*n* =16, including the three Chilean strains of this study), *C. insidiosus* (*n* = 6), *C. nebraskensis* (*n* = 4), *C. sepedonicus* (*n* = 3), *C. tessellarius* (*n* = 2), *C*. *capsici* (*n* = 1), *C. michiganensis* subsp. *phaseoli* (*n* = 1), *C. michiganensis* subsp. *californiensis* (*n* = 1), and *C. michiganensis* subsp. *chilensis* (*n* = 1), as well as 14 strains not assigned to any subspecies, were included in the ANIm analysis of these.

### 2.6. Identification of Virulence Factors, Pathogenicity Islands and Antibiotic Resistance Determinants

Protein sequences of previously determined and characterized virulence factors in strains of *C. michiganensis* subsp. *michiganensis* [9,24,36] were used as references for detection of virulence genes, using the TBLASTN tool of BLAST 2.7.1+ software [50], against local databases of genome sequences from publicly available genomes of *C. michiganensis* subsp. *michiganensis* strains and strains of other *Clavibacter* spp. isolated from tomato. Sixteen strains of *C*. *michiganensis* subsp. *michiganensis;* including the three Chilean strains of this study, *C. michiganensis* subsp. *californiensis* strain CFBP 8216^T^, *C. michiganensis* subsp. *chilensis* strain CFBP 8217^T^, four strains, *C. michiganensis* strains CFBP 7491, CFBP 7493, Z001 and Z002, that were not assigned to any subspecies, and strains of *Clavibacter* spp. that were reclassified from *C. michiganensis* subspecies, were included in the analyses. Additionally, tomato endophytic (i.e., non-pathogenic) strains, *C. michiganensis* subsp. *michiganensis* strains CFBP 8017, CFBP 8019, CFBP 7576, CFBP 7494 and CASJ009 were also included in the analysis (Table S3). Amino acid sequences of Pat-1, Chp and Php proteins (Chp family proteases), Ppa proteins (chymotrypsin-related serine proteases), CelA (cellulase), Xys (xylanases), Sbt proteins (serine proteases), PgaA (polygalacturonase), PelA1 and PelA2 (pectinases) of the reference, *C. michiganensis* subsp. *michiganensis* strain NCPPB 382 were used for gene searches. A tomatinase (TomA), a perforine (PerF) and a sortase (SrtA), which are implicated in pathogenicity, were also used for gene searches. Genomic searches for the Vatr-1 and Vatr-2 transcriptional regulators that affect disease incidence and the severity of blister formation was also assessed. Searches for two endoglucanases, encoded by the *endX/Y* genes (CMM_2691 and CMM_2692) in strain NCPPB 382, that may participate in cellulose degradation, were also performed. Pseudogenes, *chpA*, *chpB*, *chpD* and *celB*, were excluded from the analysis. Amino acid sequences were retrieved from UniProtKB-Swissprot databases [51] and the NCBI protein database (https://www.ncbi.nlm.nih.gov/). Additionally, the Pfam database, version 32.0, was used for identifications of protein families [52]. Genomic searches for *C. michiganensis* subsp. *michiganensis* pathogenicity islands, known as the *chp/tomA* region, were performed by local alignment, using the BLASTN tool of BLAST 2.7.1+ software, against a local genome sequence database of the Chilean strains, VL527, MSF322 and OP3. The TBLASTN and BLASTN outcomes were filtered, using the following cutoffs: e-value <10^–10^, identity >40%, alignment length >50%, and gap <4%. Additionally, BLAST results for each of the genes were checked manually. Results were represented in heatmaps, using pheatmap, version 1.0.8 R package [49].

Alternatively, predictions of genomic islands in the strains, VL527, MSF322 and OP3, were performed, using the tool, IslandViewer version 4 [53]. Genome sequences were aligned against the complete genome sequence of the reference, *C. michiganensis* subsp. *michiganensis* strain NCPPB 382. IslandViewer version 4 integrates different tools, including SIGI-HMM [54], IslandPath-DIMOB [55] and the comparative genomic island prediction IslandPick [56].

Genome sequences were also analyzed, with the Comprehensive Antibiotic Resistance Database (3.0.0) (CARD), using the Resistance Gene Identifier (RGI) tool, with perfect and strict hits only criteria [57].

### 2.7. Phylogenetic Analysis of Pathogenicity Genes

Phylogenetic relationships of key virulence genes (*celA*, *chpC*, *pat-1* and *pelA1*) within pathogenic strains of *C. michiganensis* subsp. *michiganensis* and endophytic strains of *Clavibacter* species were assessed, using MEGA version 7 [58]. Nucleotide sequences were extracted from the genomic sequence data of all analyzed strains and manually trimmed. Multiple sequence alignments were done, using MUSCLE [59]. Maximum-likelihood phylogenetic trees of virulence genes were constructed, based on the Tamura 3-parameter model (*celA*, *pat-1* and *pelA1*), allowing for some sites to be evolutionarily invariable [60], and the Kimura 2-parameter model for *chpC*, as proposed by Akaike Information Criterion for selection of the appropriate evolutionary model, using MEGA version 7.

### 2.8. Statistical Analysis

The data from pathogenicity assays were checked for normality and homogeneity of variances, through the Shapiro–Wilk test and the Levene test, respectively. The data then were analyzed by a one-way analysis of variance (ANOVA). The means were compared by Tukey’s HSD (honestly significant difference) test (*p* ≤ 0.05), using RStudio version 1.3.959-1 and R version 4.0.2. Data are presented as the mean values, with standard deviations. Treatments for which values for all data were zero (disease symptoms on uninoculated controls) were excluded from the statistical analyses.

## 3. Results

### 3.1. Chilean Strains Induced Symptoms of Bacterial Canker in Tomato

**Figure 1:**
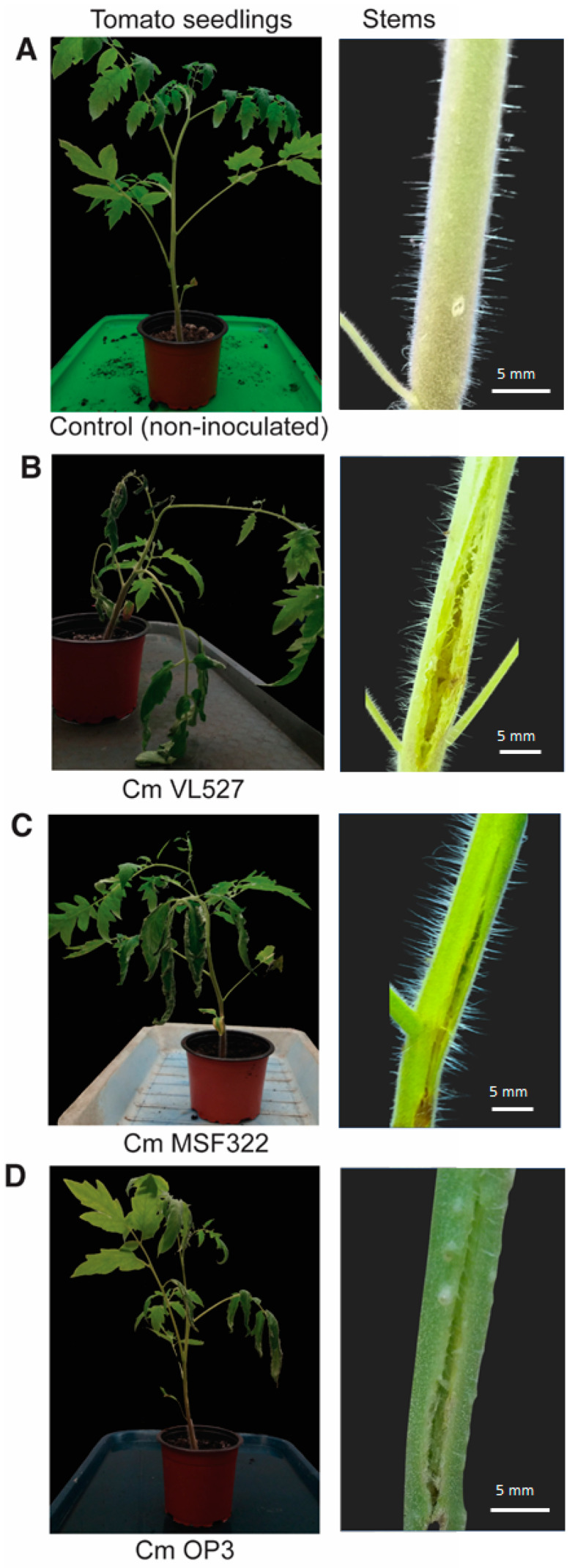
Experimental assays for pathogenicity of the Chilean strains VL527, MSF322 and OP3 in tomato seedlings. Disease symptoms of tomato seedlings 21 days after inoculation with strains VL527 (**B**), MSF322 (**C**), OP3 (**D**), belonging to three different phylogenetic clades of *C. michiganensis* subsp. *michiganensis*, and mock inoculated, non-inoculated control (**A**). Tomato seedlings were stabbed into the stems at 1 cm from cotyledons (red arrow), using a toothpick dipped in a fresh bacterial colony. A representative plant of each experiment is shown. Symptoms observed in tomato seedlings inoculated with strain VL527, exhibiting the greatest disease index (DI), were more severe, resulting in wilting of the entire plant and canker extended along the stem (**B**). Tomato seedlings inoculated with strains MSF322 or OP3 showed wilting only in some leaves and canker in the stems (**C** and **D**). Tomato plants inoculated with strain OP3 exhibited the lowest disease index (DI). No wilting and only the wound caused by the toothpick in the stem was observed in mock-infected plants.

In this study, we postulated that the Chilean *Clavibacter michiganensis* subsp. *michiganensis* strains VL527, MSF322 and OP3, that are representatives of three different phylogenetic clades, based on the previous MLSA-MLST and VNTR-MLVA studies [21], present differences in virulence for tomato plants. The three strains, VL527, MSF322 and OP3, were observed to be pathogenic to tomato seedlings. Plants showed typical symptoms of bacterial canker, including chlorosis and wilting, and canker which was initiated at the site of inoculation and extended along the stem (Figure 1). After 21 days cultivation, inoculated tomato seedlings were examined and classified according to disease symptoms. The symptoms were observed earliest and were more severe, according to the calculated disease index (DI), in plants inoculated with strain VL527 (DI value of 65.33), wherein canker extended along the stem (observed as stem cracking) and wilting of the entire plants was observed in most of the tomato seedlings (Figure 1B). The seedlings inoculated with strain MSF322 (DI value of 58.67) showed also canker extended along the stem but wilting affected only part of the plants (Figure 1C). The strain OP3 exhibited weak to moderate wilting symptoms in tomato seedlings (DI value of 48.00) (Figure 1D). Tomato seedlings inoculated with sterile water, as control, showed no wilting symptoms and only the wound caused by the toothpick in the stem was observed (Figure 1A).

Statistical analyses indicated a significant difference between the DI of strain OP3 and that of VL527. The DI of strain MSF322 was observed to be intermediate between the DI values of strains OP3 and VL527 (Table 2).

### 3.2. Whole-Genome Sequence Analysis of Strains VL527, MSF322 and OP3

#### 3.2.1. Assembly and Genome Features

**Figure 2:**
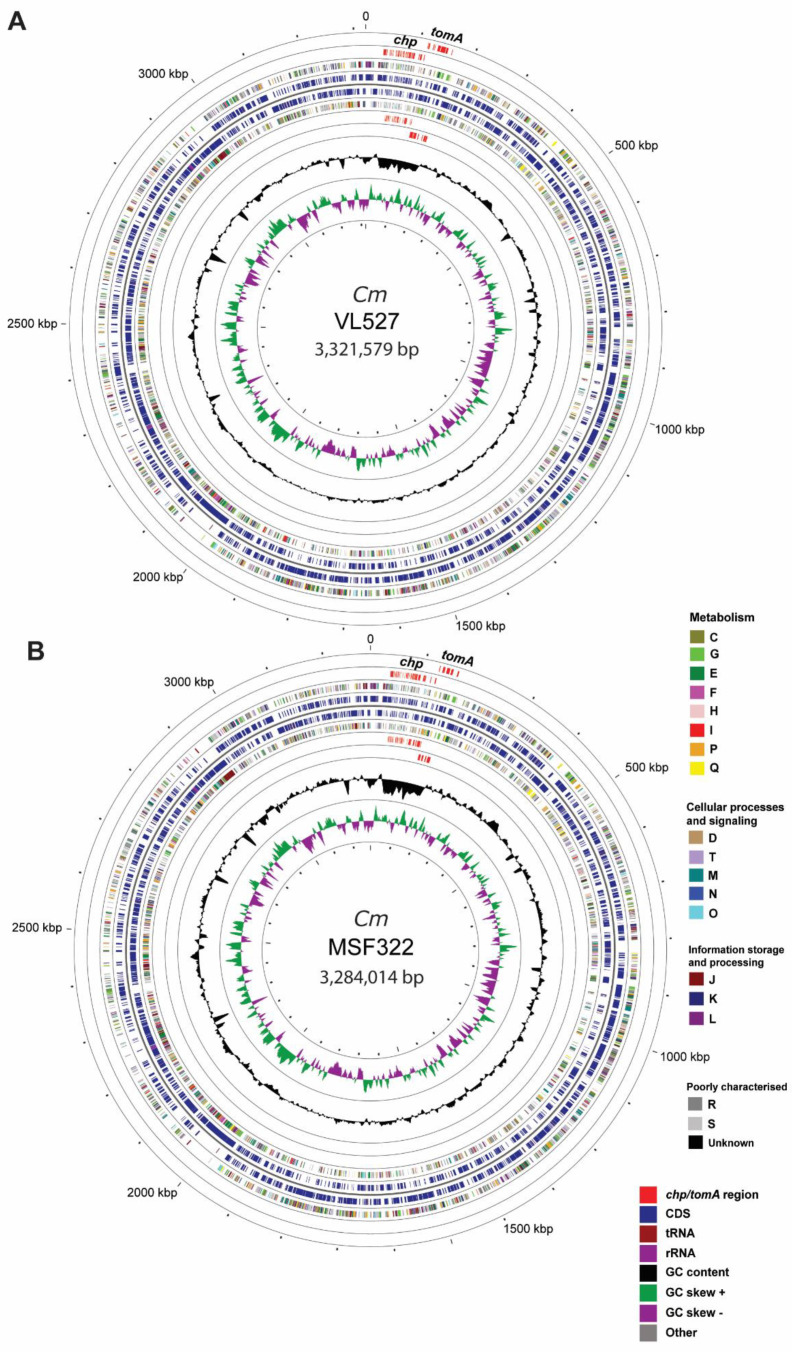
Schematic representation of the chromosomes of the Chilean strains VL527 and MSF322. The chromosome of C. *michiganensis* subsp. *michiganensis* strain VL527 has a size of 3,321,579 bp, 3067 coding sequences, 6 rRNA, and 52 tRNA (**A**); the chromosome of strain MSF322 has a size of 3,284,014 bp, 3039 coding sequences, 6 rRNA, and 53 tRNA (**B**). Rings from inside to outside: GC skew (+/−) (1); GC content (2); Predicted genes in reverse strand (4); Predicted genes in forward strand (5); predicted pathogenicity island (*chp/tomA* region) in reverse and forward strands based on BLASTN (3,6) (7). Gene function predictions were annotated, based on clusters of orthologous groups (COG) categories. The chromosome maps were generated, using CGview.

We hypothesized that Chilean strains VL527, MSF322 and OP3, representatives of three different phylogenetic clades of *C. michiganensis* subsp. *michiganensis* strains presenting different levels of plant virulence, may show differences in virulence gene content. The genomes of Chilean strains VL527, MSF322 and OP3 were sequenced, assembled, and analyzed, to characterize the genomic features, the diversity of relevant virulence genes and the phylogenetic relationships with other *C. michiganensis* strains. The yield and quality of the Illumina and Oxford Nanopore sequencing runs are presented in Supplementary Table S1. The genome assemblies of strains VL527 and MSF322 resulted in closed genomes of 3,396,632 bp and 3,399,199 bp, respectively, both with 72.6% G+C content. The genome of strain VL527 possesses a single plasmid of 75,053 bp (plasmid one; hereafter, pVL2; 67% G+C content), whereas the genome of strain MSF322 contains two circular plasmids of 38,824 bp (plasmid one; hereafter, pMSF1; 66.9% G+C) and of 76,361 bp (plasmid two; hereafter, pMSF2; 67.8% G+C content). On the other hand, the assembly of strain OP3 resulted in a genome sequence, that was not closed, of 3,466,104 bp, comprised of five contigs of 3,189,274 bp (contig one; 72.4% G+C content), 131,602 bp (contig two; 65.8% G+C content), 73,139 bp (contig three; 67% G+C content), 38,824 bp (contig four; 67.8% G+C) and 33,265 bp (contig five; 67.3% G+C). For strains VL527 and MSF322, Unicycler yielded circular contigs (contig one), which represented the chromosome, including the *chp*/*tomA* region. However, in the case of strain OP3, Unicycler Assembly yielded a contig, representing the chromosome (contig one), but that did not contain the *chp*/*tomA* region. The *chp*/*tomA* region was assembled, but in a separate contig (contig two). Two additional *de novo* assemblies were performed, using Canu and Flye, which unlike Unicycler’s hybrid strategy, are based only on Nanopore long sequence reads. Additionally, a reference-based assembly was performed, with CLC Genomics Workbench, using the Illumina reads of OP3 strain for mapping and the genome sequence of *C. michiganensis* subsp. *michiganensis* strain NCPPB 382 as a reference. In total, 14.4 million Illumina reads (91%) were mapped and the *chp*/*tomA* region was well covered, with only two gaps of 348 and 48 bp, respectively, located within central regions of the genomic island. However, the three different *de novo* assembly strategies used, assembled the *chp*/*tomA* region in a different contig, alone, separated from the rest of the chromosome, which did not allow closure of the chromosome. Genomic features of strains VL527, MSF322 and OP3 are summarized in Table 3. Circular representations of the chromosomes of strains VL527 (Figure 2A) and MSF322 (Figure 2B) are illustrated.

#### 3.2.2. Genome Annotation

Genome annotation identified 3164 (strain VL527), 3117 (strain MSF322) and 3174 (strain OP3) coding sequences (CDSs). Interestingly, one CRISPR array was identified in the genomes of strains VL527 and OP3, but not in strain MSF322 (Table 3). Chromosomal genes of strains VL527 and MSF322 were classified, based on COG nomenclature (Figure 2), using Prokka annotation. Distributions of COG categories in these two strains were highly similar. The three most abundant categories in strains VL527 and MSF322 corresponded to functions, unknown (S; 27.1 and 26.6%, respectively), carbohydrate transport and metabolism (G; 8.3% and 8.4%, respectively) and transcription (K; 6.6% and 6.5%, respectively).

The complete 16S rRNA gene sequences were extracted from the genome sequences of strains VL527, MSF322 and OP3. Sequence analysis revealed ≥99.9% similarity for the 16S rRNA gene sequences of the Chilean strains with that of *C. michiganensis* subsp. *michiganensis* strain LMG 7333^T^ (=VKM Ac-1403^T^), the type strain of *C. michiganensis* subsp. *michiganensis*.

#### 3.2.3. Plasmid Annotation

Genome characterization included the analysis of plasmids present in the Chilean strains, which are known to carry virulence genes. The plasmids of strains VL527, MSF322 and OP3 were analyzed to characterize their general features and to determine the relationships with previously reported *Clavibacter* plasmids and their relevant virulence genes. Plasmid analysis was performed, using the previously reported plasmids of *C. michiganensis* subsp. *michiganensis* strain NCPBB 382, as reference. Sequence analysis, using BLASTN, indicated that plasmid pVL2 of strain VL527 possesses 99.8% sequence identity (56% of query length) with the plasmid pCM2 of strain NCPPB 382. Similarly, the plasmid pMSF2 (76.4 kb) of strain MSF322 possesses 99.0% of sequence identity (47% of query length) to the plasmid pCM2 (70 kb) of strain NCPPB 382. However, the plasmid pMSF1 of strain MSF322 exhibits a low sequence similarity with plasmid pCM1 of strain NCPPB 382 and shows 89.0% identity (9% coverage) with plasmid pCI1 of *C. insidiosus* strain R1-3. At least three homologous sequence regions, identified, using the progressiveMauve algorithm, were detected within the larger plasmids of strains VL527, MSF322 and NCPPB 382 (Figure S1). Several genes associated with the function of plasmids were identified in these sequences. Two resolvase-encoding genes (GRD61_15865 and GRD61_16000) and an integrase-encoding gene (GRD61_16005) were detected, located in plasmid pVL2 of strain VL527. Genes encoding a relaxase (GRD61_15780) and the ParB protein (GRD61_15830) were identified in the plasmid pVL2. Genes encoding a chromosome-partitioning protein, ParA (GRD74_15675 in pMSF2 and GRD74_15915 in pMSF1) and ParB (GRD74_15580 in pMSF2) were identified in strain MSF322. Two resolvase-encoding genes are present in plasmid pMSF1 (GRD74_15975 and GRD74_16015) and two are present in plasmid pMSF2 (GRD74_15630 and GRD74_15810), whereas an integrase-encoding gene (GRD74_15815) is located downstream of the GRD74_15810 gene in plasmid pMSF2.

The hybrid assembly of strain OP3 resulted in a draft genome comprised of five contigs. In order to determine which of these contigs constitute plasmids, further sequence analyses were performed. Based on alignment analyses, strain OP3 contig one possesses 99.6% identity (coverage of 96%) with the genome of the reference *C. michiganensis* subsp. *michiganensis* strain NCPPB 382. Contig two (131,602 bp) also possesses high sequence identity (99.1% identity; 96% coverage) with the chromosome of strain NCPPB 382, matching the *chp*/*tomA* region, supporting our proposal that contig two is part of the chromosome (contig one). Contig three (73,139 bp; hereafter, pOP2) possesses 94.2% identity with the plasmid pCM2 of strain NCPPB 382 (69,989 bp). Contig four (38,824 bp; hereafter, pOP3) possesses 87.8% identity (6% coverage) with the plasmid pCM2 of *C. michiganensis* strain NCPPB 382. Interestingly, contig five (33,265 bp; hereafter, pOP1) possesses 99.4% identity (69% coverage) with the plasmid pCM1 (27,357 bp) of *C. michiganensis* subsp. *michiganensis* strain NCPPB 382. Additionally, an analysis, using the progressiveMauve algorithm, showed that contig five possesses two sequence regions homologous with the plasmid pCM1 (Figure S2A) and contig three possesses four homologous regions with the plasmid pCM2 (Figure S2B). Plasmid-associated genes are present in contigs three, four and five: three relaxase-encoding genes (GQ603_15820, GQ603_15960, GQ603_15995) and a TraM relaxosome protein-encoding gene (GQ603_16005) in contig three; two resolvase-encoding genes (GQ603_16175 and GQ603_16215) in contig four; and a resolvase gene (GQ603_16365) in contig five. These results suggest that strain OP3 possesses three plasmids, pOP1 (contig five), pOP2 (contig three) and pOP3 (contig four). Moreover, contigs three, four and five of the genome sequence of strain OP3 possess lower G+C contents (67.0%, 67.8% and 67.3%, respectively), compared with the G+C content of the largest contig (contig one), which has a G+C content of 72.9%, strongly suggesting that these contigs are derived from plasmids.

#### 3.2.4. Antibiotic Resistance Genes

To further study genetic determinants involved in bacterial defense strategies, antibiotic resistance genes were studied. Genome searches for antibiotic resistance genes, using the Comprehensive Antibiotic Resistance Database, did not detect any matches in the genomes of strains VL527 and MSF322. However, strain OP3 was observed to possess a single nucleotide polymorphism (K39R) in the *rpsL* gene, which may confer streptomycin resistance. Streptomycin resistance has been observed for strain OP3 by Valenzuela et al. [61].

### 3.3. Pathogenicity Islands in Strains VL527, MSF322 and OP3

**Figure 3:**
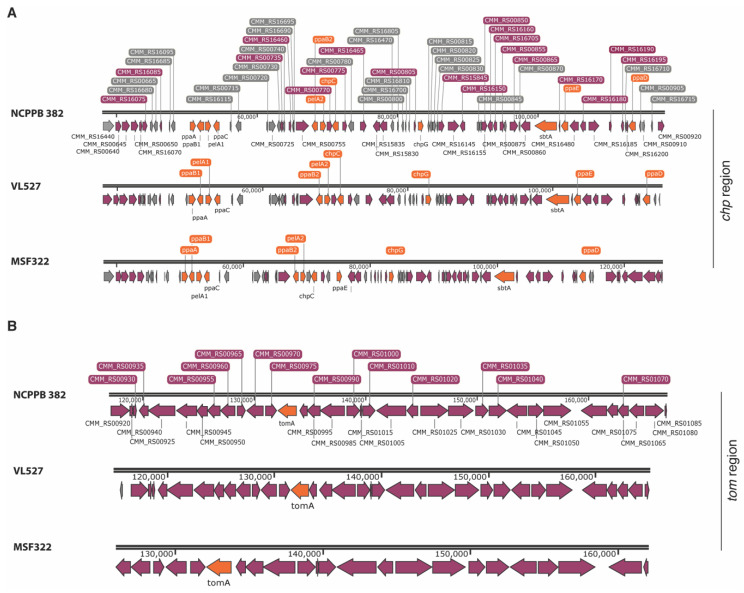
Schematic representation of the pathogenicity regions of the Chilean strains VL527 and MSF322. The *chp* (**A**) and *tom* regions (**B**) of C. *michiganensis* subsp. *michiganensis* strains NCPBB 382, VL527 and MSF322 are shown. The virulence genes (e.g., *pelA*, *chpC*, *chpG*, *ppaA—ppaE*, *sbtA*, *tomaA*) are represented in orange. The genes with other functions are depicted in purple and hypothetical genes are illustrated in grey.

The pathogenicity island (*chp/tomA* region) was detected in the chromosomes of strains VL527 and MSF322 and in a separate contig in strain OP3. The sequence of the pathogenicity island of strain NCPPB 382 was used as reference; the *chp* region is 79,050 bp, the *tomA* region is 49,650 bp, both located in the chromosome of strain NCPPB 382. A schematic representation of the *chp*/*tomA* regions of *C. michiganensis* subsp. *michiganensis* strains NCPPB 382, VL527 and MSF322 is shown in Figure 3. The *chp* region identified in the chromosome sequences of strains VL527 (GRD61_00190-GRD61_00535) and MSF322 (GRD74_00185-GRD74_00565) possesses 99.0% and 98.0% nucleotide identity, respectively, with the *chp* region of strain NCPPB 382. The *chp/tomA* regions are located near the chromosomal origins of replication of strains VL527 and MSF322, with total sizes of 132 kb and 128 kb, respectively (Figure S3). The *tomA* regions, with 99% identity with the *tomA* region of the reference strain, were identified, contiguous (downstream) to the *chp* region in strains VL527 (GRD61_00540-GRD61_00700) and MSF322 (GRD74_00570-GRD74_00675). The *chp* regions of strains VL527 and MSF322 possess average G+C contents of 64.9% and 64.7%, respectively, and the *tomA* regions of strains VL527 and MSF322 possess G+C content of 66.9% and 66.8%, respectively; the *chp* and *tomA* regions of strain NCPPB 382 possess average G+C contents of 64.8% and 66.8%, respectively [36].

Alternatively, the genomes of strains VL527, MSF322 and OP3 were analyzed for pathogenicity islands, using the genomic island prediction tool, IslandViewer version 4. All genomes were aligned against the reference strain NCPPB 382 genome. Predicted genomic islands matched with the *chp/tomA* region of strain NCPPB 382. Genomic island data obtained for strains VL527, MSF322 and OP3 are shown in Table S2. Locations of genomic pathogenicity islands in strains VL527, MSF322 and OP3 are shown in Figure S3.

As described above, we proposed that contig two of strain OP3 comprises part of the chromosome. Accordingly, contig two of strain OP3 possesses 99% identities with the *chp* (81,369 bp; GQ603_15240-GQ603_15605) and *tomA* (31,994 bp; GQ603_15145-GQ603_15235) regions of strain NCPPB 382. The 1.9 kb direct repeat sequence, flanking the *chp/tomA* region of strain NCPBB 382, is also present in contig one (at approximately position 40,000) and in contig two.

### 3.4. Pangenome Analysis of Chilean Strains

The pangenome of the Chilean *C. michiganensis* subsp. *michiganensis* strains VL527, MSF322 and OP3, and the reference strain NCPPB 382 was determined to define exclusive and shared genes of these strains that may explain observed virulence differences,. The pangenome of the Chilean strains VL527, MSF322 and OP3, as well as the reference strain NCPPB 382, was represented by 3382 genes, considering the consensus intersection between the algorithms OMCL and COGT. Of these, 49 genes were present only in the strain VL527, 43 genes were unique in the strain MSF322 and 91 genes were present only in the strain OP3. Of the exclusive genes present in strain VL527, genes encoding a peptidase (GRD61_RS13055), three carbohydrate ABC transporter permeases (GRD61_RS02135, GRD61_RS02140 and GRD61_RS02145), two LacI family transcriptional regulator (GRD61_RS00220 and GRD61_RS02125), and 21 hypothetical proteins were detected and identified. MSF322 possesses exclusively genes encoding two hydrolases (GRD74_RS03640 and GRD74_RS08035), two peptidases (GRD74_RS12885 and GRD74_RS04555), and 16 hypothetical proteins. Strain OP3 possesses exclusively genes encoding a peptidase (GQ603_RS12455), a LysR family transcriptional regulator (GQ603_RS15890) and 60 hypothetical proteins. Further function prediction analyses of hypothetical proteins, using eggNOG and Pfam servers, predicted genes encoding a third LacI family transcriptional regulator (GRD61_RS00215) in strain VL527, a peptidase (GRD74_RS11205) and a metallopeptidase (GRD74_RS03635) in strain MSF322, and a glycosyl hydrolase (GQ603_RS01130) in strain OP3. Table S3 shows the list of exclusive genes present in each of the Chilean strains, derived from the pangenome analysis.

### 3.5. Phylogenomic Placement of Strains VL527, MSF322 and OP3

**Figure 4:**
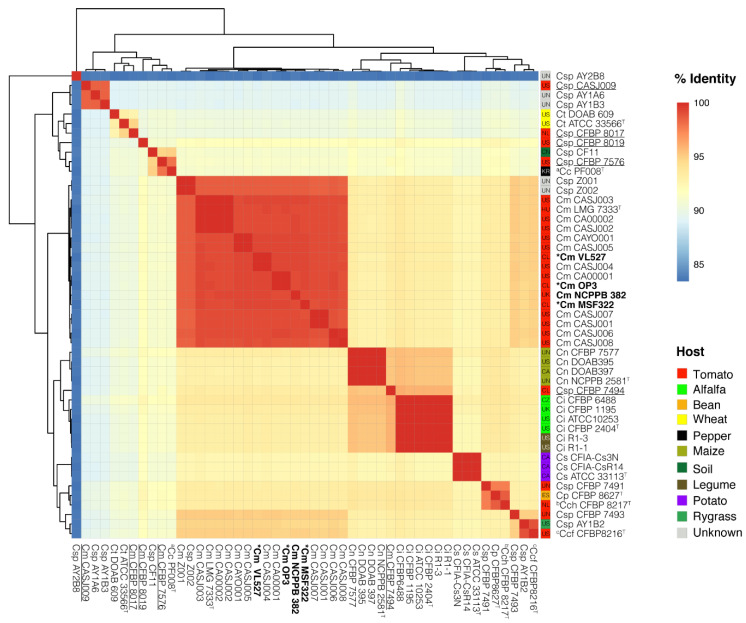
Genome–genome comparisons of representative strains of *Clavibacter* species, including the Chilean *C*. *michiganensis* subsp. *michiganensis* strains, VL527, MSF322 and OP3. Clustering based on ANIm analysis of 49 strains listed as *C. michiganensis* is shown (left). Chilean *C*. *michiganensis* subsp. *michiganensis* strains VL527, MSF322, and OP3 (bold, asterisk) are grouped within the main *C. michiganensis* cluster, together with the reference *C*. *michiganensis* subsp. *michiganensis* strain NCPPB 382 (bold). Strains of *C*. *michiganensis* subsp. *michiganensis* (Cm), *C*. *capsici* (^a^Cc), *C. michiganensis* subsp. *californiensis* (^b^Ccf), *C. michiganensis* subsp. *chilensis* (^c^Cch), *C. michiganensis* subsp. *phaseoli* (Cp), *C*. *insidiosus* (Ci), *C*. *nebraskensis* (Cn), *C*. *sepedonicus* (Cs), *C. tessellarius* (Ct) species, endophytic strains (underlined) and strains of *Clavibacter* sp. (Csp), i.e., with no designated subspecies were included in the analysis. Isolation sources of strains are indicated (right) and corresponding countries of origin are indicated before each strain name: Canada (CA), Chile (CL), China (CN), Czech Republic (CZ), Hungary (HU), South Korea (KR), Spain (ES), the Netherlands (NL), United Kingdom (UK), United States (US), and of unknown origin (UN). ANIm similarities are shown in Table S5.

In this study, we hypothesized that the Chilean VL527, MSF322 and OP3 strains belong to the subspecies, *C. michiganensis* subsp. *michiganensis*, and are closely related to the reference strain NCPPB 382 and the type strain of the species and the subspecies, LMG 7333^T^, and distinct from other subspecies, i.e., subsp. *phaseoli*, subsp. *californiensis* and subsp. *chilensis*. Genome-to-genome comparative analysis of *C. michiganensis* strains (listed in Table S4) featuring a predominant, main clade, with 18 strains of *C. michiganensis* subsp. *michiganensis* clustering with greater than 98.0% ANIm similarities with reference strain NCPPB 382 and the type strain LMG 7333^T^. This main clade, which includes strains isolated from the tomato, included the three Chilean strains VL527, MSF322 and OP3 strains from the USA (16 strains), Hungary (one strain), and the UK (one strain) (Figure 4). Strain VL527 possesses the closest phylogenetic relatedness with strain NCPPB 382, with 99.2% ANIm similarity (96.1% coverage), while strains MSF322 and OP3 possess 99.1% ANIm similarities to strain NCPPB 382 (96.2% and 96.5% coverage, respectively) (Table S5).

### 3.6. Search for Potential Virulence Markers in Strains VL527, MSF322 and OP3

**Figure 5:**
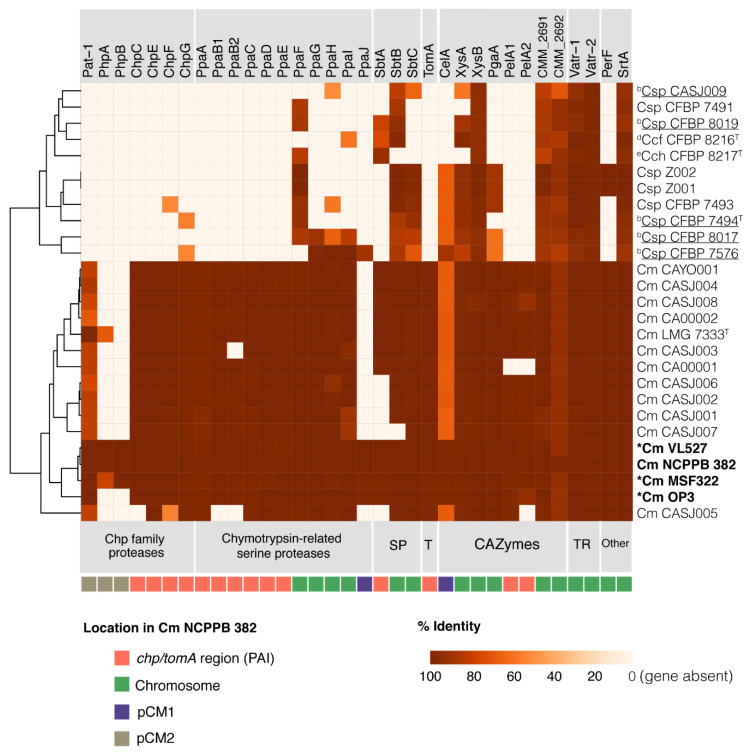
Heatmap of genes associated with virulence in *C. michiganensis* subsp. *michiganensis* pathogenic and non-pathogenic strains. Chp family proteases, chymotrypsin-related proteases, subtilase proteases (SP), tomatinase (T), carbohydrate active enzymes (CAZymes), transcriptional regulators (TR), and other virulence-related genes (Other) are shown in *C. michiganensis* subsp. *michiganensis* and endophytic strains (underlined). Heatmap shows identity (%) related to the corresponding amino acid sequences of strain NCPPB 382. Gene locations in the strain NCPPB 382 genome (PAI, chromosome, pCM1, pCM2) are indicated. Identity percentages are indicated in Table S6.

We proposed that each of the Chilean *C. michiganensis* subsp. *michiganensis* strains VL527, MSF322 and OP3 possesses most virulence genes reported in pathogenic *C. michiganensis* subsp. *michiganensis* strains and absent in endophyte strains, although they have unique repertoires of virulence genes. Additionally, we proposed that particular virulence genes of the Chilean strains VL527, MSF322 and OP3 possess variability that may explain the differences in the virulence levels in tomato plants observed in this study. In this study, we characterized the virulence genes in the Chilean strains VL527, MSF322 and OP3 to correlate the different levels of aggressiveness observed in tomato plants. Additionally, a genomic search and comparative analysis was performed with twelve other pathogenic *C. michiganensis* subsp. *michiganensis* strains and with five non-pathogenic endophytic strains. According to our phylogenomic analysis, strains Z001 and Z002 belong to *C. michiganensis* subsp. *michiganensis*; therefore, they were included in the analysis, as well as four other strains (CFBP 7491, CFBP 7493, CFBP 8217^T^ and CFBP 8216^T^) that were isolated from the tomato, although the phylogenomic analysis showed the strains to be only distantly related to *C. michiganensis* strains. Figure 5 shows sequence identities of translated genes found in all strains analyzed, related to virulence factors described in strain NCPPB 382.

#### 3.6.1. Chp Family Proteases

Most of known virulence genes have been observed to be present among all pathogenic strains and are highly conserved. However, amino acid sequences of the plasmid-encoded members of the Chp family proteases are variable within pathogenic strains. The Pat-1, PhpA, PhpB encoding genes exhibited the highest sequence variability within pathogenic strains, with nucleotide sequence identities ranging from 40–100% (Figure 5, Table S6). The *pat-1* gene products of strains VL527 and MSF322, whose genes are located in the plasmids pVL2 and pMSF2, respectively, possess greater than 99% amino acid sequence identities with the Pat-1 protein of strain NCPPB 382, as well as the *pat-1* gene product of strain OP3 (Figure 5, Table S6). A homologue of the *phpA* gene product of strain MSF322 exhibited lower amino acid sequence identity (79%) with the PhpA protein of strain NCPPB 382, whereas PhpA of VL527 showed 100% identity with the NCPPB 382 PhpA protein (Figure 5), while strain OP3 does not possess the *phpA* and *phpB* genes. Other members of the Chp family proteases located in the *chp/tomA* region, the *chp* gene products of *C. michiganensis* subsp. *michiganensis* strains including the Chilean strains, possess high amino acid sequence identities with the ChpC, ChpE, ChpF, and ChpG proteins of NCPPB 382 (>99% identity).

#### 3.6.2. Chymotrypsin-Related Serine Proteases

Chymotrypsin-related serine proteases (*ppa* genes) exhibited high similarities at the protein level within all pathogenic strains (Figure 5). Sequences of the PpaA, PpaB1, PpaB2, PpaC, PpaD, and PpaE proteins of all pathogenic strains, including Chilean strains, showed higher than 98.0% identities with the proteins of strain NCPPB 382. The *ppaJ* gene products of Chilean strains VL527, MSF322 and OP3 share higher than 99.0% amino acid sequence identities with the PpaJ protein of strain NCPPB 382.

#### 3.6.3. Subtilase Proteases and Tomatinase

Subtilase proteases (SP), which are encoded by the *sbtA*, *sbtB* and *sbtC* genes, exhibited low sequence variability within pathogenic strains, whereas higher variability was observed in non-pathogenic strains. The presence of the *sbtA* gene, located in the *chp/tomA* region of strain NCPPB 382, is present in Chilean strains VL527, MSF322 and OP3 and variable within all analyzed strains.

The *sbtB* and *sbtC* gene products of the Chilean strains VL527, MSF322 and OP3 are conserved, possessing sequence identities greater than 99% with the Sbt proteins of strain NCPPB 382. The *tomA* (tomatinase) gene product homologues are highly conserved, with greater than 99% identities across all pathogenic strains, including the Chilean strains VL527, MSF322 and OP3. However, the tomatinase-encoding *tomA* gene was not found in the non-pathogenic strains analyzed (Figure 5).

#### 3.6.4. CAZymes

All genes, except the *celA* gene, encoding carbohydrate active enzymes (CAZymes) are highly conserved in pathogenic strains. Chilean strains VL527, MSF322 and OP3 possess genes encoding proteins with >99.0% identities with the CelA protein of strain NCPPB 382. The *celA* gene was identified in the pVL2 and pMSF2 of strains VL527 and MSF322, respectively, and in the pCM1-like plasmid (pOP1) of strain OP3. Genes encoding the pectinase PgaA (polygalacturonase) are highly conserved across all strains of *C. michiganensis* (>99% identity), including the Chilean strains VL527, MSF322 and OP3. Two pectate lyases encoded by the *pelA1* and *pelA2* genes were found in all pathogenic *C. michiganensis* strains, including the Chilean strains VL527, MSF322 and OP3.

Amino acid sequences of xylanases, encoded by *xysA* and *xysB* genes, are highly conserved among all strains analyzed, particularly XysB. However, the *xysA* gene products of the endophytic strains possess the lowest sequence identities (58–93%) with the XysA protein of strain NCPPB 382, among all *Clavibacter* species. The *xysA* gene is absent in *C. michiganensis* subsp. *chilensis* strains CFBP 8217^T^ (Figure 5). Two endoglucanases encoded by *endX/Y* (CMM_2691, CMM_2692 in strain NCPPB 382) are highly conserved among all strains analyzed, although, the CMM_2692 gene product is less conserved (70–93% identities).

#### 3.6.5. Transcriptional Regulators, Perforine and Sortase

The *perF* gene, encoding a perforine, is highly conserved among pathogenic *C. michiganensis* subsp. *michiganensis* strains, whereas the gene is absent in endophytic strains. The transcriptional regulators Vatr-1 and Vatr-2, and the SrtA protein (sortase) are highly conserved in all *C. michiganensis* subsp. *michiganensis* strains.

### 3.7. Genetic Relationships of Virulence Genes in C. michiganensis

**Figure 6:**
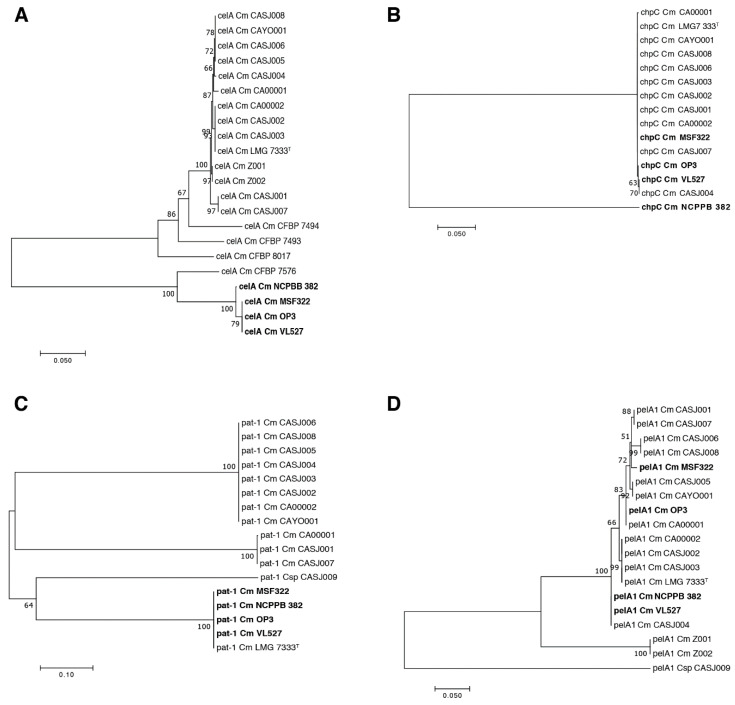
Phylogenetic analysis of *celA*, *chpC*, *pat-1* and *pelA1* genes. Phylogenetic relationships of *celA* (**A**), *pat-1* (**C**) and *pelA1* (**D**) genes were inferred by the maximum likelihood method, based on the Tamura 3-parameter. The rate variation model allowed for some sites to be evolutionarily invariable (39.8% sites). The analysis involved 20 nucleotide sequences. There was a total of 1302 nucleotide positions in the final dataset. Analysis of the *chpC* gene (**B**) was inferred by the maximum likelihood method, based on the Kimura 2-parameter model. Methods were proposed by Akaike information criterion for selection of appropriate evolutionary model using MEGA version 7. The trees are drawn to scale, with branch lengths measured in the number of substitutions per site.

Phylogenetic relationships of the *celA*, *chpC*, *pat-1* and *pelA1* genes were assessed to further characterize key genetic determinants for pathogenicity in the Chilean strains VL527, MSF322 and OP3. The *celA* genes of strains VL527, MSF322 and OP3 group in a monophyletic clade, together with the *celA* gene of *C. michiganensis* subsp. *michiganensis* strain NCPPB 382 and other pathogenic strains (Figure 6A). The *chpC* gene of Chilean strains VL527, MSF322 and OP3 and other pathogenic strains clustered in a monophyletic clade, except for the gene of strain NCPPB 382, which comprises a singleton (Figure 6B). As shown in Figure 6C, three distinguishable clusters of the *pat-1* gene were observed. The *pat-1* gene of strains VL527, MSF322, OP3, NCPPB 382 and LMG 7333^T^ appeared as a monophyletic group. The *pelA1* and *pat-1* genes of the endophytic strain CASJ009 are distantly related to the *pelA1* and *pat-1* genes of other *C. michiganensis* pathogenic strains, conforming singletons. The *pelA1* gene of strains VL527, MSF322 and OP3 grouped in three different clusters (Figure 6D), showing that the *pelA1* gene of strain VL527 is closely related to the *pelA1* gene of strains NCPPB 382 and CASJ004. The *pelA1* gene of MSF322 is closely related to *pelA1* genes of strains CASJ006 and CASJ008, while the pelA1 gene of strain OP3 clustered with *pelA1* of strain CA00001.

## 4. Discussion

In a previous study, we analyzed the emergence and dissemination of *C. michiganensis* subsp. *michiganensis* in Chile, by the characterizing twenty-five strains, including the three strains analyzed in this study, isolated from different locations in central Chile [21]. MLSA and MLST, based on five housekeeping genes, and multi-locus VNTR analysis (MLVA), based on eight VNTRs, clustered the Chilean strains VL527, MSF322 and OP3 in three phylogenetic clusters within the *C. michiganensis* subsp. *michiganensis*, each corresponding to a single sequence type [21]. In the present study, pathogenicity assays confirmed that strains VL527, MSF322 and OP3 effect differences in virulence levels in tomato plants (Figure 1). In this study, we proposed to characterize genetic determinants to account for the different virulence levels observed in Chilean *C. michiganensis* subsp. *michiganensis* strains VL527, MSF322 and OP3, which may be used as genetic markers for future virulence studies. Different virulence levels and specificity for tomato plants as host may be explained by genomic differences, such as the presence/absence of virulence genes. For example, endophytic strains do not possess the pathogenicity island (*chp/tomA* region) but are able to colonize tomato plants [18]. Occurrence of non-pathogenic *C. michiganensis*-like bacteria in tomato plants is frequent and of increasing concern for seed producers. Therefore, reliable strain detection and identification should be addressed in order to avoid elimination of healthy seeds. Accordingly, high genomic variability is expected to occur among non-pathogenic and pathogenic strains.

A genomic analysis of the *chp/tomA* pathogenicity islands in Chilean strains, revealed that strains VL527, MSF322 and OP3 possess a *chp/tomA* region with high similarity to the *chp/tomA* region of the reference pathogenic strain NCPPB 382. Occurrence of the *chp/tomA* genomic island, reported to be essential for pathogenicity, is found in the genomes of all pathogenic *C. michiganensis* subsp. *michiganensis* strains but not in the non-pathogenic strains [18,36]. Previous studies of several *C. michiganensis* subsp. *michiganensis* strains, including strain NCPPB 382, reported that gene products involved in infection, colonization and suppression of plant defences are encoded in the chromosome [36]. The highly conserved *chp/tomA* region, located in the chromosome of strain NCPPB 382 and other *C. michiganensis* subsp. *michiganensis* strains, contains genes encoding proteases and CAZYmes essential for effective colonization and expression of disease symptoms in tomato plants [17,18]. Non-pathogenic *C. michiganensis*-like bacteria lack the *ppaJ*, *chpC*, *tomA*, *ppaA* and *ppaC* genes, which are located in the PAI [36]. The virulence factors encoded in the *chp/tomA* region in strain NCPPB 382 are highly conserved among the Chilean strains VL527, MSF322 and OP3 and other *C. michiganensis* subsp. *michiganensis* pathogenic strains, whereas the PAI-encoded genes are absent in non-pathogenic strains (Figure 5). Although the *chp/tomA* region is highly conserved in the Chilean strain OP3, this strain exhibited the lowest expression of disease symptoms in tomato seedlings. Therefore, other genes and mechanisms, and changes in gene sequences and regulation may be relevant for the strongest plant symptoms observed when inoculated with strains MSF322 and VL527.

Bacterial plasmids play an important role in virulence and acquisition of genetic traits for environmental adaptation. Accordingly, a variable number of plasmids among strains of *C. michiganensis* subsp. *michiganensis* has been reported [18]. Endophytic strains do not possess plasmids with similarities to pCM1 or pCM2 from *C. michiganensis* subsp. *michiganensis* strain NCPPB 382 [18]. The Chilean *C. michiganensis* subsp. *michiganensis* strains of this study possess a variable number of plasmids. Strain VL527 possesses a single plasmid, strain MSF322 possesses two plasmids, and strain OP3 possesses three plasmids (Figure S2). Plasmid sequences alignment analyses indicated that plasmids pVL2, pMSF2 and pOP2 resemble plasmid pCM2 of reference, *C. michiganensis* strain NCPPB 382, in which several homologous regions were identified (Figure S1). Since pCM1 and pCM2 of strain NCPPB 382 are conjugative plasmids, reacquisition and loss of plasmids can be expected within a bacterial population but maintaining key genes for virulence and host specificity. Plasmids pCM1 and pCM2 of strain NCPPB 382 carry the virulence *celA* and *pat-1* genes, respectively. These genes are present at different genomic locations in the Chilean *C. michiganensis* strains. In strains MSF322 and VL527, the *celA* and *pat-1* genes are located in the pCM2-like plasmid (pMSF2 and pVL2), whereas in strain OP3 the *celA* and *pat-1* genes are located in the pOP1 (pCM1-like plasmid) and pOP2 (pCM2-like plasmid), respectively. Interestingly, amino acid sequences of the *celA* and *pat-1* gene products of strains VL527, MSF322 and OP3, are highly conserved compared to the CelA and Pat-1 proteins of strain NCPPB 382. The *celA* and *pat-1* genes have been reported to have a critical role in disease induction, wilting, and canker symptoms in the tomato. Gene expression of the *celA* gene encoding an endoglucanase in non-virulent strains induces wilt of tomato plants [62]. Although some strains of *C. michiganensis* subsp. *michiganensis* naturally lack pCM2-like plasmids [18], the *pat-1* gene is one of the most important virulence genes [63]. Introduction of the *pat-1* gene into a plasmid-cured non-virulent *C. michiganensis* subsp. *michiganensis* strain NCPPB 382 derivative, restored virulence [64]. Some strains that lack pCM2-like plasmid still are pathogenic to the tomato to a similar level as strains that possess pCM2 [18]. Strains VL527 and OP3 possesses identical Pat-1 amino acid sequences relative to strain NCPPB 382, whereas Pat-1 of strain MSF322 differs in only one amino acid (Figure 5). Along with the *pat-1* gene, the *phpA* and *phpB* genes are members of the Chp family that are also located in the plasmid pCM2 of strain NCPBB 382. The *phpA* and *phpB* genes are also located in pCM2-like plasmids of strains VL527 and MSF322. In contrast, the *phpA* and *phpB* genes were not present in strain OP3 and in non-pathogenic strains (Figure 5). Previous studies reported that the *phpA* gene is upregulated in minimal medium containing plant tissue homogenate, but not the *phpB* gene [65]. Conversely, Burger et al. [66] determined that homologues of *phpA* and *phpB* genes do not induce disease symptoms in tomato plants. All Californian strains lack these genes and exhibit different levels of virulence [18]. These studies suggest that the *phpA* and *phpB* genes are not essential for pathogenicity.

Other relevant chromosomally encoded virulence factors have been characterized in *C. michiganensis*. Members of the Chp family proteases, located on the *chp/tomA* region of strain NCPPB 382, were observed to be highly conserved in Chilean strains VL527, MSF322 and OP3 and other pathogenic strains. The *chpC*, *chpD*, *chpE*, *chpF* and *chpG* genes, located in the *chp/tomA* region in *C. michiganensis* strains, encode proteins with amino acid sequences identical to those of strain NCPPB 382 (Figure 5). Homologues of the *chp* genes were not found in endophytic strains, except for the *chpG* gene that is present in strains CFBP 7494 and CFBP 7576 (Figure 5 and Table S6). Up-regulation of the *chpC*, *chpE*, *chpF*, and *chpG* genes, and induction of the corresponding proteins has been observed in infected tomato plants [67]. In addition, up-regulation of the *chpA*, *chpC*, *chpF* and *chpG* genes was detected in minimal medium with tomato homogenate [65], whereas the *chpC* gene was also induced during foliar infection [24]. Thus, the *chp* genes are likely to possess a relevant role in pathogenicity mechanisms. Accordingly, the *chpC*–*chpG* gene cluster was not present in non-pathogenic strains (Figure 5). However, their specific roles and function have not been described.

Other virulence factors, such as chymotrypsin-related serine proteases (*ppa* genes) are highly conserved in Chilean strains VL527, MSF322 and OP3 and other pathogenic strains, except the *ppaB2* and *ppaJ* gene products (Figure 5). The *ppa* genes, located in the *chp/tomA* region of strain NCPPB 382, are absent in non-pathogenic strains. The *ppaF*, *ppaG*, *ppaH*, and *ppaI* genes, located outside the *chp/tomA* region in the chromosome, are conserved among pathogenic strains and non-pathogenic strains (Figure 5). However, the *ppaA*–*ppaE* genes are more conserved than the *ppaF*–*ppaJ* genes, which is in agreement with their location in the highly conserved *chp/tomA* region in strain NCPPB 382. Most pathogenic *C. michiganensis* and non-pathogenic strains lack the *ppaJ* gene. Interestingly, Chilean strains VL527, MSF322 and OP3 possessed almost identical amino acid sequences to the PpaJ protein of strain NCPPB 382. The *ppaJ* gene is located on the plasmid pCM1 of strain NCPPB 382 [36]. Likewise, the *ppaJ* gene was identified on the plasmids pVL2, pMSF2 and pOP2 of strains VL527, MSF322 and OP3. Endophytic strains possess some of the *ppa* genes in the chromosome, outside the *chp/tomA* region. The *ppaF* gene was present in almost all analyzed strains, except in strains CASJ009 (endophyte), CFBP 8216^T^ (*C. michiganensis* subsp. *californiensis*), and CFBP 7576. The *ppaJ* gene along with the chromosomally encoded genes *ppaA*, *ppaB1, ppaB2*, *ppaC*, *ppaD* and *ppaH*, were upregulated in a minimal medium containing tomato homogenate [65]. The induction of PpaB1, PpaB2, PpaC, PpaD, PpaH and PpaI proteins in infected tomato plants [67] suggest that these chymotrypsin-like serine proteases may possess a key role in virulence. In addition, no up-regulation of the *ppaF*, *ppaG* and *ppaI* genes was observed in minimal medium containing tomato homogenate [65].

The *tomA* gene encodes a tomatinase, which is involved in the degradation of the glycoalkaloid tomatine that provides basal defense in tomato against pathogens [68,69]. The TomA encoding gene, also located in the *chp/tomA* region of strain NCPPB 382, is highly conserved in Chilean strains VL527, MSF322 and OP3 and other pathogenic strains but absent in non-pathogenic strains (Figure 5). Studies performed with fungal pathogens of tomato plants suggest that the *tomA* gene is required for full virulence but is not essential for virulence [70].

Pectinases include two pectate lyases encoded by the *pelA1* and *pelA2* genes, located in the *chp/tomA* region of strain NCPPB 382, and a polygalacturonase that is encoded by the *pgaA* gene, located outside the PAI in the chromosome. Of all the genes encoding CAZymes, only the *pelA1* and *pelA2* genes are present in Chilean strains VL527, MSF322 and OP3 and all other pathogenic strains. Except for the strain CASJ009, endophytic and non-pathogenic strains do not possess the *pelA1* and *pelA2* genes. The *pgaA* gene was present in Chilean strains VL527, MSF322 and OP3 and all other pathogenic strains but also in non-pathogenic strains CFBP 7493, Z001 and Z002, and in endophytic strains CFBP 8017 and CFBP 7576 (Figure 5). Up-regulation of the *pgaA* gene was observed in minimal medium containing tomato homogenate [65], whereas a *pgaA* gene mutant reduced incidence of foliar blisters in tomato plants [24].

To further clarify genomic traits that may be related to different pathogenicity levels observed within Chilean strains, phylogenetic trees were constructed for four virulence genes that significantly affect disease incidence, i.e., *chpC*, *pat-1, celA* and *pelA1* genes (Figure 6). Interestingly, the most polymorphic gene was the *pelA1* gene encoding a pectate lyase. The *pelA1* genes from the Chilean strains clustered in three different groups (Figure 6D). The strain VL527 grouped with strain NCPPB 382 and the Californian strain CASJ004. These results, and the absence of this gene in endophytic strains, suggest that polymorphism of the *pelA1* gene may play a role in the different virulence levels observed for Chilean *C. michiganensis* subsp. *michiganensis* strains VL527, MSF322 and OP3. The pectate lyase, PelA1, has been described to be important for the development of pathogenic symptoms. Chalupowicz et al. [71] reported that *pelA1* gene expression increased during the first 24 h post inoculation. Tomato plants infected with *C. michiganensis* CASJ002 strain, lacking the *pelA1* gene, exhibited reduced wilting, compared with plants infected with wild type, whereas no differences were observed in plants infected with a *pelA2* mutant strain [18]. Interestingly, the phylogenetic clustering observed from comparison of *pelA1* gene sequences in Californian strains (Figure 6D), is in accordance with analyses based on orthologous genes [18], except for strains CASJ004 and CA00001. Some correlations also were observed for virulence levels between strains [18], and clustering of *pelA1* genes. Some strains that grouped together in the *pelA1* phylogenetic tree, such as strains CASJ006 and CASJ008, strains NCPPB 382 and CASJ004, and strains CA0002 and CASJ003, presented similar percentages of wilted plants. However, unexpectedly, the *pelA1* gene of the Chilean strain OP3, exhibiting the lowest degree of virulence among the Chilean strains, clustered with virulent strain CA00001 [18]. Interestingly, plasmids pCM1 and pCM2 exert a slight but significant negative effect on *pelA1* gene expression [71].

Low genetic diversity of *chpC* gene among the *C. michiganensis* subsp. *michiganensis* strains VL527, MSF322 and OP3 and other pathogenic strains was observed. All *chpC* genes revealed a monophyletic origin, except the *chpC* gene of strain NCPPB 382. The *chpA*, *chpB, chpC, chpD, chpE, chpF* and *chpG* genes are located within the *chp/tomA* region in the PAI. The presence of the *chpC* gene has been related to colonization of bacteria and may be critical for virulence [69]. CelA and Pat-1, two proteins described as key factors for virulence, exhibited high genetic diversity among *C. michiganensis* strains (Figure 5). However, Chilean strains VL527, MSF322 and OP3 possessed identical *pat-1* and *celA* genes, although differing from those of strains from California [18]. The *pat-1* genes of strains VL527, MSF322, OP3 and NCPPB 382 clustered in the same phylogenetic clade. The *celA* genes of Chilean strains VL527, MSF322 and OP3 also clustered with the *celA* gene of strain NCPPB 382 (Figure 6C). Conversely, the *celA* genes of non-pathogenic strains CFBP 7576, CFBP 8017, CFBP 7494 and CFBP 7493 did not cluster with their homologues in pathogenic strains. The analysis of the virulence genes of this study and previous reports suggest a complex interplay and regulation of different virulence gene products for bacterial canker virulence in tomato plants.

In this study, we proposed to define the phylogenomic positions of strains VL527, MSF322 and OP3 among *Clavibacter* spp. A genome–genome comparative analysis of *C. michiganensis* subsp. *michiganensis* strains VL527, MSF322 and OP3, and 46 other strains identified as *Clavibacter* species was carried out, including the type strains from all validly published species of the genus, *Clavibacter*, and the subspecies of the species, *C. michiganensis*. The three Chilean strains, VL527, MSF322 and OP3, exhibited their phylogenomic placement within the predominant *C. michiganensis* subsp. *michiganensis* clade, which grouped together 16 strains isolated from tomato plants in the USA, Hungary, UK, and two strains from unknown sources sharing high ANIm similarities (>98.5%) (Figure 4). These results indicate that all strains within this clade belong to the *C. michiganensis* species, in accordance with the proposed genomic similarity boundary for species delineation (ANI value >95.0%) [72]. Chilean strains VL527, MSF322 and OP3 shared high ANIm similarities (>99.0%) to the pathogenic *C. michiganensis* subsp. *michiganensis* strain NCPPB 382, which correlates with previous taxonomic classifications, based on MLSA-MLST, using five housekeeping genes, and VNTR-MLVA, using eight VNTRs [21]. *Clavibacter* sp. strains Z001 and Z002, unclassified at the species (or subspecies) level, of unknown hosts and geographic locations, were phylogenetically placed within the *C. michiganensis* subsp. *michiganensis* clade (Figure 4). Strains CASJ004 and CA00001 were observed to be closely related to the Chilean strains, showing >99.0% ANIm similarities (Figure 4). Accordingly, the strains CASJ004 and CA00001, isolated from tomato plants, have been previously classified as strains of *C*. *michiganensis* subsp. *michiganensis*. As expected, other strains included in the genome–genome analysis, classified as *Clavibacter* species (*C. insidiosus, C. sepedonicus, C. nebraskensis, C. tessellarius, C. capsici*) that were formerly classified as subspecies of *C. michiganensis* were observed to group in distinct and separate clades.

Non-pathogenic endophytic strains (CASJ009, CFBP 7494, CFBP 7576, CFBP 8017, and CFBP 8019), also isolated from tomatoes, were placed in clades with strains of different species (Figure 4), indicating greater genetic diversity among them than was observed among the pathogenic strains. Strain CFBP 7576, a non-pathogenic endophyte, isolated from the tomato and unclassified at the species or subspecies levels, clustered outside the main *C. michiganensis* subsp. *michiganensis* clade (Figure 4); strain CFBP 7576 was observed to be closely related to the species *C. capsici* strain PF008 (97.9% ANIm similarity, 84% alignment coverage), isolated from a pepper plant in South Korea (Table S5). Other endophytic strains, isolated from tomato plants in the USA (CFBP 8017 and CFBP 8019), did not cluster with the main *C. michiganensis* subsp. *michiganensis* clade (Figure 4). Strains CFBP 8017 and CFBP 8019 exhibited ANIm similarities between 90.1–90.3% and 91.7–91.9%, respectively, with the strains of the *C. michiganensis* subsp. *michiganensis* clade (average 75% alignment coverage). Strains CFBP 8017 and CFBP 8019 exhibited 94.8% and 90.3% ANIm similarities, respectively, with the type strain, ATCC 33566^T^, of the species, *C. tessellarius*, pathogenic to wheat (Figure 4). Similarly, strain CASJ009 clusters outside the main *C. michiganensis* subsp. *michiganensis* clade, possessing 89.2–89.4% ANIm similarities with the strains of this cluster, with an average alignment coverage of 72.0%. Strain CASJ009 was observed to be closely related (98.5% ANIm similarities) to the strains AY1A6 and AY1B3, of unknown origin (Figure 4). Previous analyses determined that endophytic strains are phylogenetically related to other subspecies (i.e., *C. insidiosus*, *C. tessellarius* and *C. capsici*) rather than to *C. michiganensis* species [18]. Analysis of eleven strains of *Clavibacter* sp. strains, including endophytic strains, CFBP 7494, CFBP 7576, CFBP 8017, CFBP8019 and CASJ009, revealed genomic diversity among them [18]. These findings indicate that genomically diverse strains of *Clavibacter* sp. can also colonize tomato plants.

Three other strains of *Clavibacter* sp. that are unclassified at the species level, CFBP 7491 (isolated from tomato plants), CFBP 7493 (isolated from tomato plants) and AY1B2 (isolated from ryegrass), were determined to be highly related to strains of *C. michiganensis* subsp. *phaseoli* and subsp. *californiensis.* Strain CFBP 7491 was found to be phylogenetically related (97.5% ANIm similarity) to the *C. michiganensis* subsp. *phaseoli* type strain, CFBP 8627^T^ (Figure 4), indicating that CFBP 7491 is a strain of that subspecies. The type strain, CFBP 8217^T^, of C. *michiganensis* subsp. *chilensis* clusters within the same ANIm similarity group of *C. michiganensis* subsp. *phaseoli* type strain (98.6%). Strains CFBP 7493 and AY1B2, unclassified at the species level, were observed to be phylogenetically related (98.8% ANIm similarity) to the type strain, CFBP 8216^T^, of *C. michiganensis* subsp. *californiensis* (Figure 4). Of these, all strains were isolated from tomato plants, except for strains AY1B2 (ryegrass) and CFBP 8627^T^ (bean), originating from different locations (the USA, Spain, The Netherlands) or of unknown locations (CFBP 7491 and CFBP 7493).

Two distinct clusters were observed for strains of the *C. insidiosus* and *C. nebraskensis* species (Figure 4). Strains NCPPB 2581^T^, CFBP 7577, DOAB 395, and DOAB 397, isolated from maize, from different geographic locations, grouped together; they had been previously classified as *C. michiganensis* subsp. *nebraskensis* species (Table S3). Similarly, six strains of *C. insidiosus* clustered together, sharing 100% ANIm similarities. Of these, four strains (CFBP 6488, CFBP 1195, CFBP 2404^T^, and ATCC 10253) were isolated from alfalfa, whereas strains R1-1 and R1-3 were isolated from a legume at different locations (Table S3). Similarly, the potato pathogenic strains, CFIA-Cs3N and CFIA-CsR14, as well as the type strain of *C. sepedonicus*, ATCC 33113^T^, clustered together, sharing 100% ANIm similarity (Figure 4, Table S5).

Strain AY2B8, included in this analysis, is listed in the public databases as *C. michiganensis*. However, our phylogenomic results showed very low ANIm similarities with the other strains included in this analysis (83–84%). Moreover, 16S rRNA gene sequence analyses indicated that the most closely related species is *Agria pratensis*. ANIm similarity between strain AY2B8 and the type strain of *A. pratensis* is 86.9%, indicating that strain AY2B8 is misclassified in public databases.

As shown in Figure 4, the ANIm similarities between many subspecies are low, generally below the 96.0% cutoff value suggested for species delineation [72]. This observation is in agreement with those described by Li et al. [4], wherein some *C. michiganensis* subspecies were reclassified and validly published at the species level; based on ANIm similarities, in silico DNA–DNA hybridization and MLSA data, the establishment of new species *C. insidiosus*, *C. nebraskensis*, *C. sepedonicus*, *C. tessellarius* and *C. capsici* was proposed. The ANIm analyses of this study suggest reclassifying *C. michiganensis* subsp. *phaseoli*, as well as *C. michiganensis* subsp. *chilensis*, at the species level, as *C. phaseoli* sp. nov., comb. nov. Based on the ANIm analyses, *C. michiganensis* subsp. *californiensis* could be reclassified to species level, i.e., as *C. californiensis* sp. nov., comb. nov. Indeed, Osdaghi et al. [73] also proposed to elevate the *C. michiganensis* subsp *phaseoli,* subsp. *chilensis* and subsp. *californiensis* to two separate *Clavibacter* species.

## 5. Conclusions

This study determined the genome sequences of three tomato pathogenic bacterial strains, VL527, MSF322 and OP3, isolated from three regions in central Chile, that represent three different *C. michiganensis* subsp. *michiganensis* phylogenetic clades, in order to correlate different observed virulence levels with genomic information. Additionally, genome comparisons enabled us to elucidate the phylogenomic placements of the three Chilean pathogenic *Clavibacter* strains and other previously unclassified strains, at the species level, within the main *C. michiganensis* phylogenetic clade that clusters together pathogenic strains of tomato plants. Comparative analysis of virulence genes showed high conservation of virulence factors in pathogenic strains. Conversely, other less conserved chromosome encoded virulence factors (outside PAI), were typically also present in non-pathogenic strains; therefore, they may not possess a critical role in pathogenicity. Although the Chilean strains VL527, MSF322 and OP3 possessed low genomic diversity among them, different virulence levels in tomato plants were observed. This may be related to key virulence genes, such as the *pelA1* gene of the *chp/tomA* region and genes encoding proteases and hydrolases that were exclusive for each of the Chilean strains.

## Figures and Tables

**Table 1:** Taxonomy of Clavibacter michiganensis.

*C. michiganensis* Subspecies (Date)	Type Strain	Disease		Host		Reclassification (Date)	Reference
*C. michiganensis* subsp. *michiganensis* (1984)	LMG 7333^T^ (=NCPPB 2979^T^)		Canker		Tomato *(Solanum lycopersicum)*		[2,5,9]
*C. michiganensis* subsp. *insidiosus* (1984)	CFBP 2404^T^ (=LMG 3663 = NCPPB 1109^T^)		Wilt and stunting		Alfalfa/lucerne *(Medicago sativa)*	*C. insidiosus* (2018)	[4,5,10]
*C. michiganensis* subsp. *nebraskensis* (1984)	NCPPB 2581^T^		Wilt and leaf blight		Maize/corn *(Zea mays)*	*C. nebraskensis* (2018)	[4,5,11]
*C. michiganensis* subsp. *sepedonicus* (1984)	ATCC 33113^T^ (=NCPPB 2137^T^)		Ring-rot		Potato *(Solanum tuberosum)*	*C. seponicus* (2018)	[4,5,12]
*C. michiganensis* subsp. *tessellarius* (1984)	ATCC 33566^T^ (=NCPPB 3664^T^)		Mosaic disease		Wheat *(Triticum aestivum)*	*C. tessellarius* (2018)	[4,5,13,14]
*C. michiganensis* subsp. *phaseoli* (2014)	CFBP 8627^T^ (=LMG 27667^T^)		Leaf yellowing		Bean *(Phaseolus vulgaris)*	*C. phaseoli* (2020)	[6], this study
*C. michiganensis* subsp. *californiensis* (2015)	CFBP 8216^T^		Asymptomatic		Tomato *(Solanum lycopersicum)*	*C. californiensis* (2020)	[8,15,16], this study
*C. michiganensis* subsp. *chilensis* (2015)	CFBP 8217^T^		Asymptomatic		Tomato *(Solanum lycopersicum)*	*C. phaseoli* (2020)	[8,15,16], this study
*C. michiganensis* subsp. *capsici* (2016)	PF 008^T^ (=LMG 29047^T^)		Canker		Pepper *(Capsicum* spp.)	*C. capsici* (2018)	[4,7,15,16]

**Table 2:** Disease indices in tomato plants inoculated with Chilean *C. michiganensis* subsp. *michiganensis* strains.

Inoculum	* Disease Index (DI)
strain VL527	65.33 ± 2.31 ^b^
strain MSF322	58.67 ± 4.62 ^a,b^
strain OP3	48.00 ± 8.00 ^a^
Uninoculated (control)	0

* Data are expressed as mean ± standard deviation (*n* = 3). Means with different letters (a,b) indicate significant differences from each other (*p* ≤ 0.05). Values obtained from the uninoculated control (zero) were excluded from the analyses of variance.

**Table 3:** Genomic features and assembly statistics for the whole-genome sequences of *C. michiganensis* subsp. *michiganensis* strains VL527, MFS322, and OP3.

^a^ Features	VL527	MSF322	OP3
GenBank Acc. No.	CP047054–CP047055	CP047051–CP047053	WTCS00000000
Finishing quality	Closed genome	Closed genome	Draft
Sequencing platforms	Illumina + Oxford Nanopore	Illumina + Oxford Nanopore	Illumina + Oxford Nanopore
Assembler	Unicycler (v0.4.7)	Unicycler (v0.4.7)	Unicycler (v0.4.7)
Annotation pipeline	PGAP (v4.10)	PGAP (v4.10)	PGAP (v4.10)
N50 (bp)	3,321,579	3,284,014	3,189,274
Chromosome (bp)	3,321,579	3,284,014	3,189,218
Plasmids	75,053 bp (pVL2)	38,824 bp (pMSF1); 76,361 bp (pMSF2)	
Contigs (No., bp)			5 contigs; Contig 1, 3,189,274 Contig 2, 131,602 Contig 3, 73,139 (pOP2) Contig 4, 38,824 (pOP3) Contig 5, 33,265 (pOP1)
Total length (bp)	3,396,632	3,399,199	3,466,104
G + C content (%)	72.6	72.6	72.4
CDS	3,164	3,117	3,174
Pseudogenes (total)	41	45	55
rRNA	2 × 5S, 2 × 16S, 2 × 23S	2 × 5S, 2 × 16S, 2 × 23S	2 × 5S, 2 × 16S, 2 × 23S
tRNA	46	45	45
CRISPR arrays	1	0	1

^a^ Genome features according to the Prokaryotic Genome Annotation Pipeline (PGAP). CDS: coding sequences.

## Supplementary Materials

The following are available online at https://www.mdpi.com/2076-2607/8/11/1679/s1, Figure S1: Sequence alignment of pCM2, pMSF2 and pVL1 plasmids of *C*. *michiganensis* strains. Figure S2: Sequence alignments of contig three and contig five of strain OP3 against plasmids of strain NCPPB 382. Figure S3: Circular representation of predicted genomic island in *C. michiganensis* strains. Table S1: Results of the Illumina and Oxford Nanopore sequencing runs. Table S2: Genomic island analysis in *C. michiganensis* strains VL527, MSF322 and OP3, Table S3: Exclusive genes present in strains VL527, MSF322 and OP3 derived from pangenome analysis. Table S4: Strains of *C. michiganensis* species used for phylogenomic analysis. Table S5: ANIm values of strains of *C. michiganensis* species. Table S6: Identity percentages of virulence genes of strains of *C. michiganensis* subsp. *michiganensis*, subsp. *californiensis*, subsp. *chilensis* and endophytic strains.
