# Supplementary material for: Comparative Genomics of Pathogenic Clavibacter michiganensis subsp. michiganensis Strains from Chile Reveals Potential Virulence Features for Tomato Plants
Source: Microorganisms. 2020 Oct 29;8(11):1679. doi: 10.3390/microorganisms8111679 (PMC7692107; doi:10.3390/microorganisms8111679)
Supplement: Supplementary file 1 [file microorganisms-08-01679-s001.zip › MendezV2020_Suppl_Material.docx]

Supplementary Material

1. Supplementary Figures


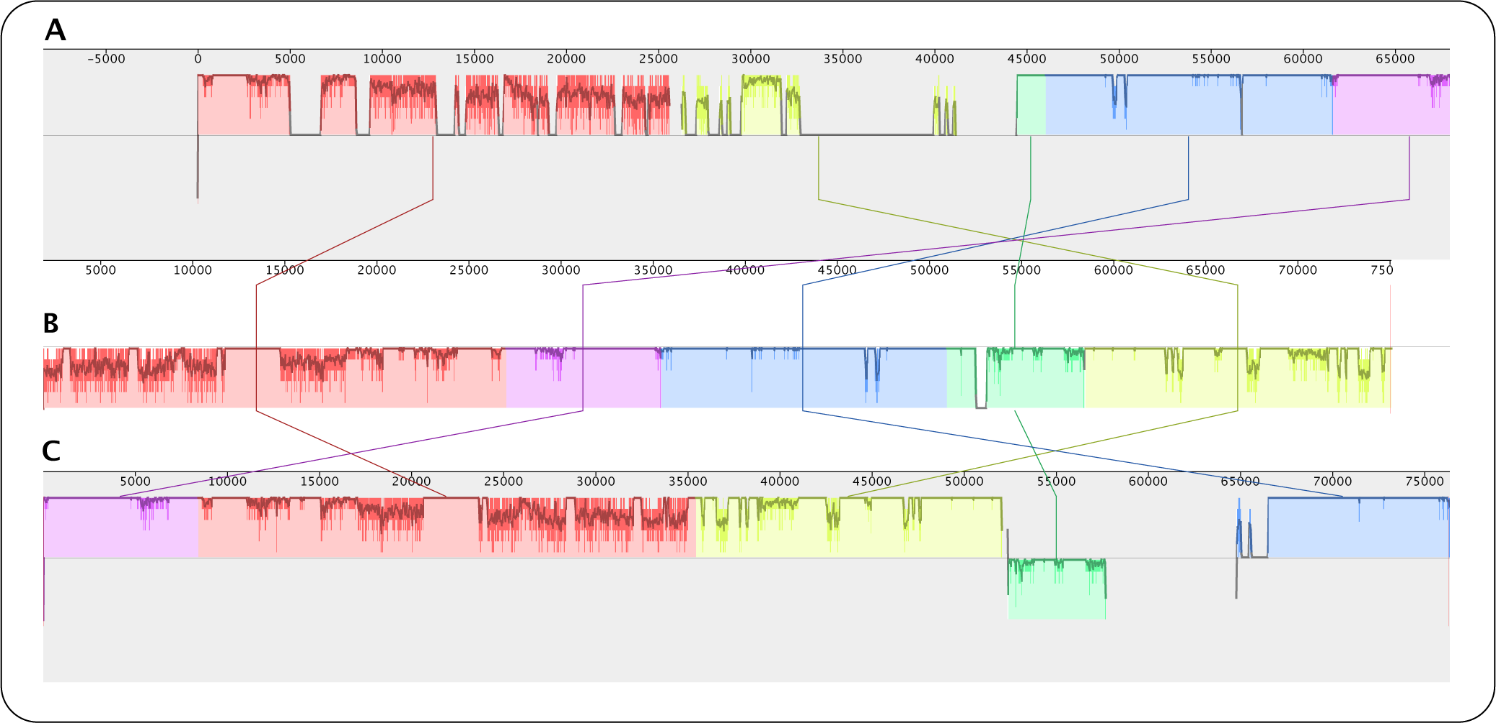


**Figure S**1. Sequence alignment of pCM2, pMSF2 and pVL1 plasmids of *C.* *michiganensis* subsp. *michiganensis* strains. Multiple nucleotide alignments were done, using progressiveMAUVE with iterative refinement and default seed weight. Alignments (from top to bottom) of pCM2 plasmid of strain NCPBB382 (A), pVL1 plasmid of strain VL527 (B) and pMSF2 plasmid of strain MSF322 (C) are shown.


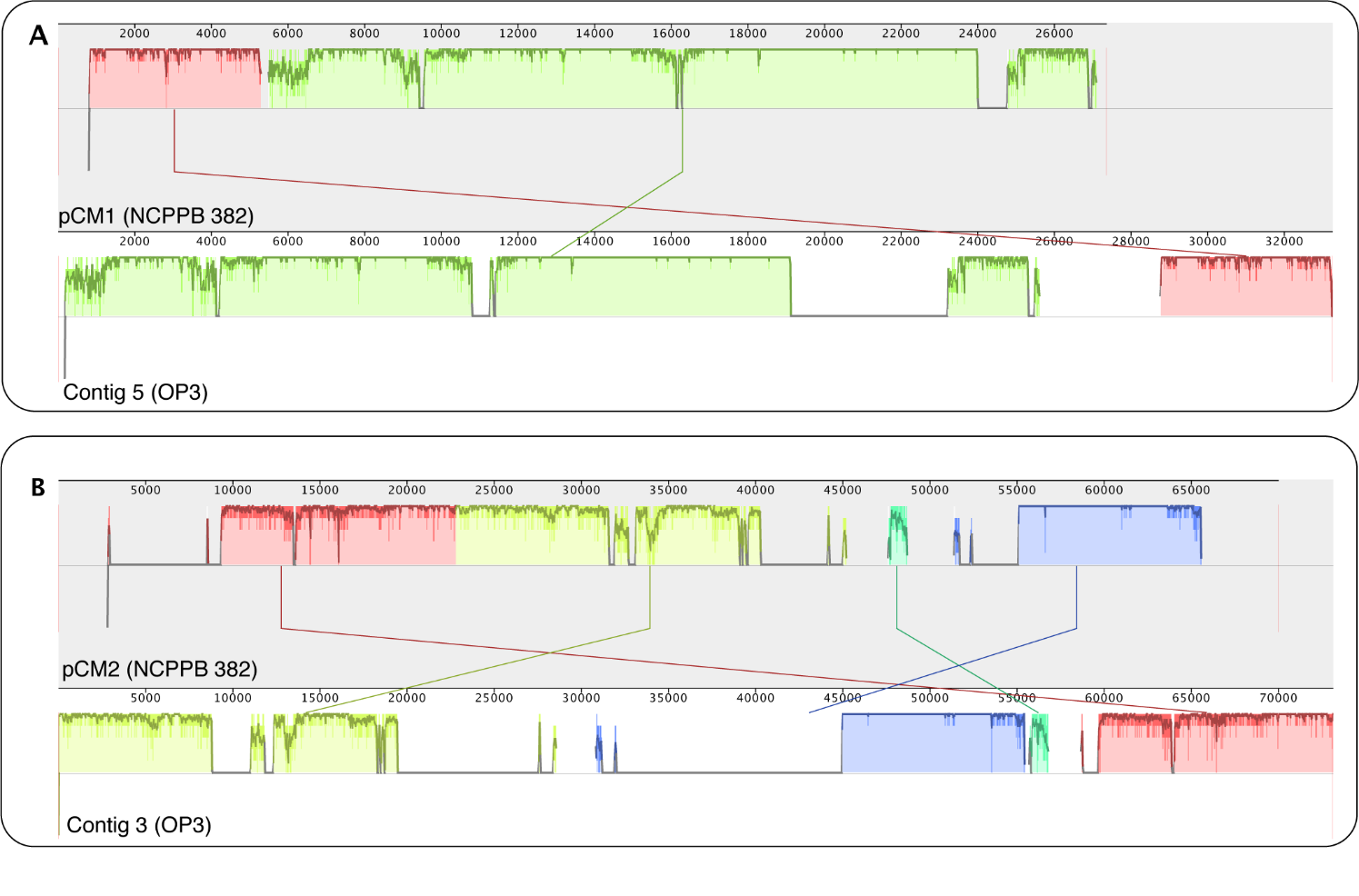


**Figure S2.** Sequence alignments of contig 3 and contig 5 of *C. michiganensis* subsp. *michiganensis* strain OP3 against plasmids of strain NCPPB 382. Contig 5 shows homologous regions with pCM1 plasmid (A), and contig 3 shows homology regions with plasmid pCM2 (B) of strain NCPPB 382. Multiple nucleotide alignments were done, using progressiveMAUVE algorithm with iterative refinement and default seed weight.


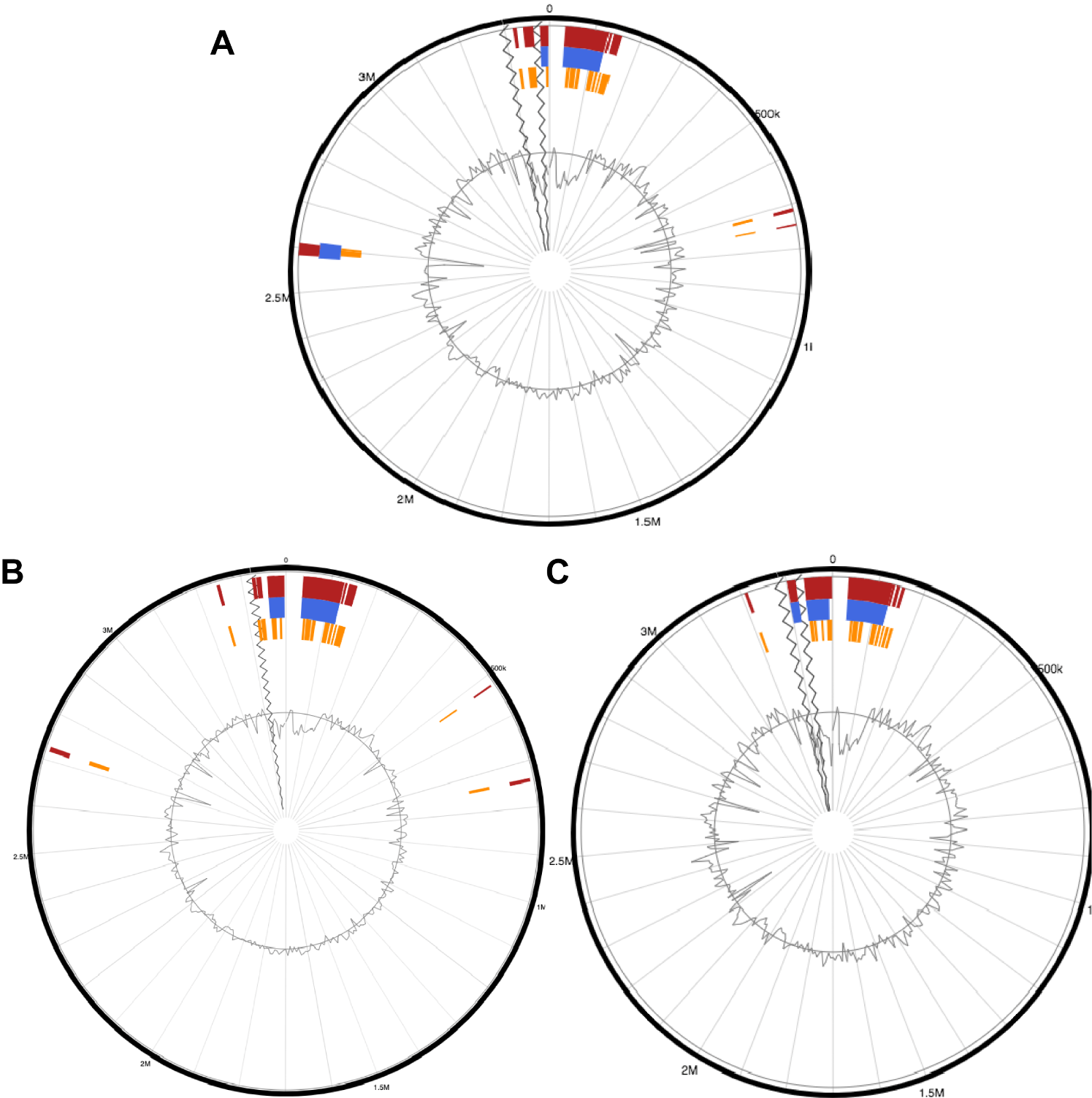


**Figure S3.** Circular representation of predicted genomic island in *C. michiganensis* subsp. *michiganensis* strains. Genomic islands against reference strain NCPPB 382 (A) were predicted in strains VL527 (B), and MSF322 (C) using Island Viewer version 4, with different tools (SIGI-HMM and IslandPath-DIMOB; orange and blue, respectively). Integrated results are shown (red). .
